# Study on the impact of multiple factors on 4-year physical fitness test results of undergraduate students at a Chinese comprehensive university: based on the mediating role of exercise habits

**DOI:** 10.3389/fpubh.2026.1819826

**Published:** 2026-04-28

**Authors:** Li Lian, Hongwei Liu, Ruijie Zhang, Guanghui Yang

**Affiliations:** 1 College of Physical Education, Beijing Wuzi University, Beijing, China; 2 College of Physical Education, Jilin Normal University, Siping, Jilin, China; 3 Department of Physical Education, Tangshan Normal University, Tangshan, China; 4 School of physical education, Yanshan University, Qinhuangdao, China

**Keywords:** college students’ physical fitness and health (PFCI), exercise habits, longitudinal study, mediating effect, structural equation modeling

## Abstract

This study employed a 4-year longitudinal tracking design with 1,154 undergraduate students at a comprehensive university in northern China as research subjects, and systematically explored the pathways through which multiple factors are associated with physical fitness and health via exercise habits using structural equation modeling and cross-lagged panel analysis. The research findings revealed: the Physical Fitness Composite Index (PFCI) decreased from 73.26 points at enrollment to 68.52 points at graduation, while exercise habit intensity decreased from 4.82 to 3.54 over the same period; exercise habits served as a statistically significant partial mediator in the process through which individual factors (self-efficacy, exercise motivation, health beliefs), social factors (peer support, family influence), and environmental factors (facility accessibility, time management) were associated with physical fitness and health, with indirect effects accounting for 39.2–60.9% of the total effects; exercise self-efficacy was the strongest individual predictor (*β* = 0.46), while time management pressure showed a significant negative association (*β* = −0.21); initial exercise habit intensity was significantly associated with long-term changes in physical fitness and health, with the high exercise habit group showing an annual average decline of 0.94 points, significantly lower than the 2.27 points in the low exercise habit group; the second semester of sophomore year and the first semester of junior year were identified as critical turning points for the decline in exercise habits. Grade-level moderation analysis further revealed that the predictive strength of exercise habits on PFCI increased progressively across academic years, and that peer support emerged as the dominant predictor during the critical transition periods. The research results suggest that stable exercise habits may represent an important factor associated with college students’ physical fitness and health levels, and that the construction of systematic approaches from multiple dimensions including individual cognitive reinforcement, social support cultivation, and environmental condition optimization may warrant consideration, with particular attention to targeted interventions during critical periods and differentiated support for high-risk groups.

## 1 Introduction

College students are at a critical period of transition from adolescence to adulthood. Physical fitness and health levels not only affect their current quality of study and life but also have a profound impact on future career development and quality of life. The four-year undergraduate education stage provides an important time window and environmental support for cultivating lifelong exercise habits. The healthy behavior patterns formed by students during this stage often extend throughout their entire life cycle into adulthood. However, the physical fitness and health status of college students worldwide shows a worrying downward trend, with problems such as significantly reduced physical activity levels, increased sedentary behavior, and loss of weight management control becoming increasingly prominent. This phenomenon is not confined to a single academic discipline. Although some studies have specifically documented the challenges faced by medical students due to their particularly demanding curricula (1), research has indicated that students across various fields—including science and engineering, liberal arts, and arts—also encounter substantial academic pressure and time constraints that may similarly hinder regular engagement in physical exercise (2, 3). Therefore, the decline in physical fitness and health appears to be a widespread issue among college students regardless of disciplinary background, though the degree and contributing factors may vary.

In recent years, international scholars have conducted extensive research on factors associated with college students’ physical fitness and health. Tang et al. (1) found through a four-year longitudinal tracking of 634 medical students that physical fitness indicators showed a significant downward trend, and there was a statistically significant correlation between BMI changes and career development paths. Based on a cross-sectional study of 3,202 college students, Wang et al. (4) confirmed that exercise demand and satisfaction play a mediating role in the transformation process from exercise behavior to exercise habits, with satisfaction accounting for 28.95% of the mediating effect. Multiple longitudinal studies have shown that exercise self-efficacy and gender factors jointly moderate the relationship between exercise motivation and physical activity (5). The formation of exercise habits requires regular exercise at least four times a week for six consecutive weeks (6). Cognitive ability and physical fitness levels play a partial mediating role in the relationship between physical activity duration and academic performance, with obvious nonlinear characteristics (7). The mediating effect of life satisfaction in the process through which physical exercise is associated with physical and mental health is moderated by social class (8). Although these studies have explored the potential mechanisms and correlates of physical fitness and health from different perspectives, most adopt cross-sectional designs or short-term tracking, lacking systematic observation of the complete university cycle, and pay little attention to the role mechanism of exercise habits as a core mediating variable in the process through which multiple factors are associated with physical fitness and health.

**Figure 1:**
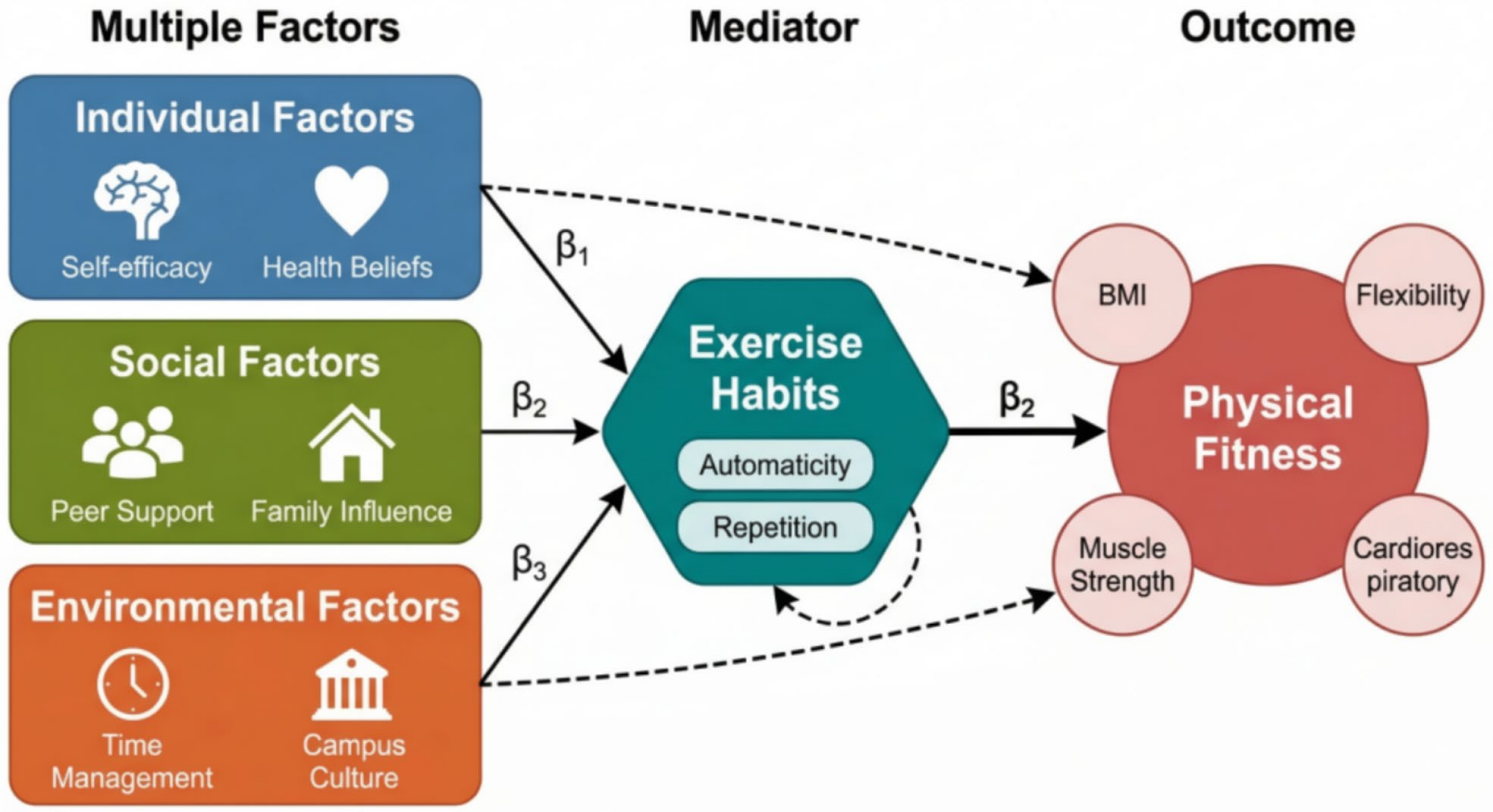
Theoretical framework of individual, social, and environmental factors affecting college students’ physical fitness and health through exercise habits.

This study adopts a four-year longitudinal tracking design to systematically collect physical fitness and health test data of college students from enrollment to graduation, constructing a theoretical framework of individual factors (self-efficacy, exercise motivation, health beliefs), social factors (peer support, family influence), and environmental factors (facility accessibility, time management) → exercise habits (frequency, intensity, persistence) → physical fitness and health outcomes (BMI, cardiopulmonary function, muscle strength, flexibility). Using structural equation modeling and Bootstrap method to test the mediating effect of exercise habits and its moderating mechanism, the innovations of this study lie in three aspects: first, expanding exercise habits from simple behavioral frequency to a composite concept including two dimensions of automaticity and repetition; second, using longitudinal cross-lagged panel analysis to examine the temporal precedence and predictive relationships rather than simple correlations between variables; and third, integrating multi-level influencing factors to construct a comprehensive theoretical model, as shown in Figure 1. This framework not only considers direct effect paths but also identifies indirect effects generated through exercise habits and interactions between different factors, providing empirical evidence for formulating targeted health promotion strategies for college students.

## 2 Literature review and theoretical framework

### 2.1 Research on factors affecting college students’ physical fitness and health

College students’ physical fitness and health are subject to complex influences from multiple levels of factors. Social determinants of health theory emphasizes that macro factors such as educational environment, economic stability, and social support networks affect individual health status through multiple pathways. These conditions not only directly affect physical health but also produce far-reaching effects through psychological mechanisms and behavioral patterns (9). Recent research has found that physical fitness and health problems among college students present new characteristics: a study covering 3,600 college students showed that myopia rates increased from 84.31% in 2018 to 89.17% in 2020, showing a year-by-year increasing trend, and insufficient physical exercise was significantly correlated with the degree of myopia. Another study showed that overweight and obesity rates among college students increased from 22.53% in 2019 to 29.25% in 2021, and the “failing” rate of physical fitness and health increased from 9.19 to 12.94%, reflecting the severe situation of college students’ physical fitness and health (3). Individual-level factors include psychological variables such as self-efficacy, exercise motivation, and body image perception. Social cognitive theory points out that self-efficacy, as a core cognitive mechanism, regulates physical activity levels by affecting individuals’ behavioral choices, effort levels, and persistence (10). Environmental-level factors cover campus facility accessibility, peer support, time management pressure, etc. A systematic review of the theoretical domain framework shows that college students’ physical activity is jointly affected by three core domains: ability, opportunity, and motivation, with environmental and resource factors occupying an important position (2).

### 2.2 Formation mechanism and measurement of exercise habits

The formation of exercise habits involves the transformation process from conscious behavior to automated response. The extended model of the theory of planned behavior introduces exercise commitment as a bridge variable between intention and behavior. Research shows that exercise commitment plays a key role in the process from behavioral intention to behavioral execution among college students, significantly improving the model’s predictive power (11). Exercise habits contain two core dimensions: repetition and automaticity. Large-sample research based on 3,202 college students confirms that exercise behavior can develop into exercise habits through the single mediation of demand and satisfaction or the chain mediation of demand → satisfaction, with satisfaction accounting for 28.95% of the mediating effect (4). Habit formation requires specific frequency and duration. Longitudinal research shows that regular exercise at least four times a week for six consecutive weeks is the basic condition for forming stable exercise habits, and the exercise frequency in the initial stage has a significant predictive effect on subsequent habit maintenance (12). The Self-Report Habit Index for exercise serves as a standardized measurement tool, quantifying habit strength by assessing the degree of behavioral automation and repetition frequency. This scale has shown good reliability and validity across different cultural backgrounds (13).

### 2.3 Mediation effect theory and application

Mediation effect analysis provides an important theoretical and methodological framework for understanding complex causal relationships. In the field of physical activity research, cognitive ability and physical fitness levels have been confirmed to play a partial mediating role in the relationship between exercise duration and academic performance, exhibiting nonlinear characteristics (7). Psychological resilience, as an important mediating variable, plays a significant role in the pathway through which physical exercise influences adolescents’ self-efficacy, with structural equation modeling analysis showing that both direct and indirect effects reach statistical significance levels (14). The mediating effect of life satisfaction in the process by which physical exercise influences physical and mental health is moderated by social class, indicating that mediation mechanisms may vary by group characteristics (8). The interaction between exercise self-efficacy and gender factors in the relationship between exercise motivation and physical activity reveals the complexity of multiple mediation, with the direct effect of competence motivation accounting for 41.5% of the total effect and the mediating effect of exercise self-efficacy accounting for 58.5% (5). The application of Bootstrap methods and structural equation modeling has made the testing of complex mediation effects more precise, and longitudinal cross-lagged panel designs can effectively identify causal directions between variables (15). The Theory of Planned Behavior provides an important framework for understanding the relationship between exercise intention and behavior, with attitudes, subjective norms, and perceived behavioral control jointly determining behavioral intention (16). A stable longitudinal relationship exists between habits and physical activity behavior, with habit strength being an important predictor of the persistence of future exercise behavior (17).

Grit, as a personality trait, plays an important moderating role in the relationship between self-efficacy and physical activity, with this moderating effect showing clear gender differences and being more prominent in male groups (18, 19). The operationalized application of Social Cognitive Theory across different cultural contexts has confirmed the universality of this theoretical framework, with German population studies validating the joint predictive effects of self-efficacy, outcome expectations, and social support on physical activity (20). The pathways through which exercise motivation influences college students’ self-efficacy are complex and diverse, with intrinsic motivation enhancing self-efficacy through strengthening exercise commitment, while the role of extrinsic motivation is relatively limited (18, 19). Physical exercise not only enhances physical self-efficacy but also promotes improvements in academic and social self-efficacy through transfer effects (21). Exercise enjoyment as an affective experience dimension, together with exercise self-efficacy, constitutes a dual motivational mechanism for physical activity participation, with their interaction effect explaining a greater proportion of variance in physical activity levels (22).

Social support influences college students’ physical and mental health through complex chain mediation mechanisms, with self-efficacy and physical activity playing serial mediating roles in the relationship where social support alleviates the relationship between negative body image and depressive symptoms (23). Sedentary college student populations have specific cognitive barriers and attitudinal issues regarding physical activity participation, with qualitative research revealing that time pressure, lack of motivation, and insufficient social support constitute a triple barrier to this group’s participation in physical exercise (24). While the Theory of Planned Behavior has been widely applied in health behavior prediction, it still has theoretical limitations in explaining the intention-behavior gap, requiring further theoretical reflection and model refinement (25). Experimental research provides more rigorous evidence for the causal relationship between exercise intention and actual behavior (26). While meta-analytic research generally supports the predictive validity of the Theory of Planned Behavior, it also reveals significant heterogeneity in prediction effects across different types of health behaviors (27). Integrating the Theory of Planned Behavior with Self-Determination Theory can provide a more comprehensive behavioral prediction framework, with the inclusion of autonomous motivation significantly enhancing the model’s explanatory power (28). Self-Determination Theory emphasizes the central position of intrinsic motivation, autonomy, and competence in the long-term maintenance of exercise behavior (29). The formation of the intention-behavior gap involves multiple psychological mechanisms, including lack of implementation intentions, self-regulation failure, and insufficient situational cues (30). Some scholars have even proposed moving beyond the traditional Theory of Planned Behavior framework to explore new theoretical perspectives for understanding the complexity of health behavior (31). The trend toward simplification in habit measurement reflects researchers’ pursuit of balancing measurement efficiency with precision, with simplified habit scales maintaining good reliability and validity while improving practicality (32).

### 2.4 Research hypotheses and conceptual model

Based on an integrated framework combining the socio-ecological model and behavior change theory, this study proposes a theoretical model in which multi-level influencing factors are associated with physical fitness through exercise habits. Three levels of factors are included in this model: individual factors (self-efficacy, exercise motivation, health beliefs), social factors (peer support, family influence, teacher encouragement), and environmental factors (facility accessibility, time resources, campus culture) constitute the multi-factor system examined in this study. It should be noted that the socio-ecological model also recognizes the importance of policy-level factors (such as physical education curriculum design and health promotion programs) in shaping health behaviors. However, in the present study, all participants were enrolled at the same university and were subject to the same institutional policies throughout the four-year observation period. As a result, policy factors functioned as constants rather than variables with measurable individual-level variation, and were therefore not included in the empirical analysis. Exercise habits, serving as the core mediating variable, connect influencing factors and health outcomes through two dimensions of automaticity and repetition, forming a hypothesized pathway of “multiple factors → exercise habits → physical fitness.”

Based on theoretical analysis and empirical research, the following research hypotheses are proposed: H1: Individual-level factors (self-efficacy, exercise motivation, health beliefs) positively predict the formation of exercise habits. H2: Social-level factors (peer support, family influence) are associated with physical fitness outcomes through the mediating role of exercise habits. H3: Environmental-level factors (facility accessibility, time management) moderate the relationship between exercise habits and physical fitness. H4: Exercise habits play a partial mediating role in the process through which multiple factors predict physical fitness, with the strength of mediation effects potentially varying by demographic variables such as gender, grade level, and disciplinary background. H5: In the four-year longitudinal tracking data, the stability of exercise habits predicts long-term trends in physical fitness changes.

**Figure 2:**
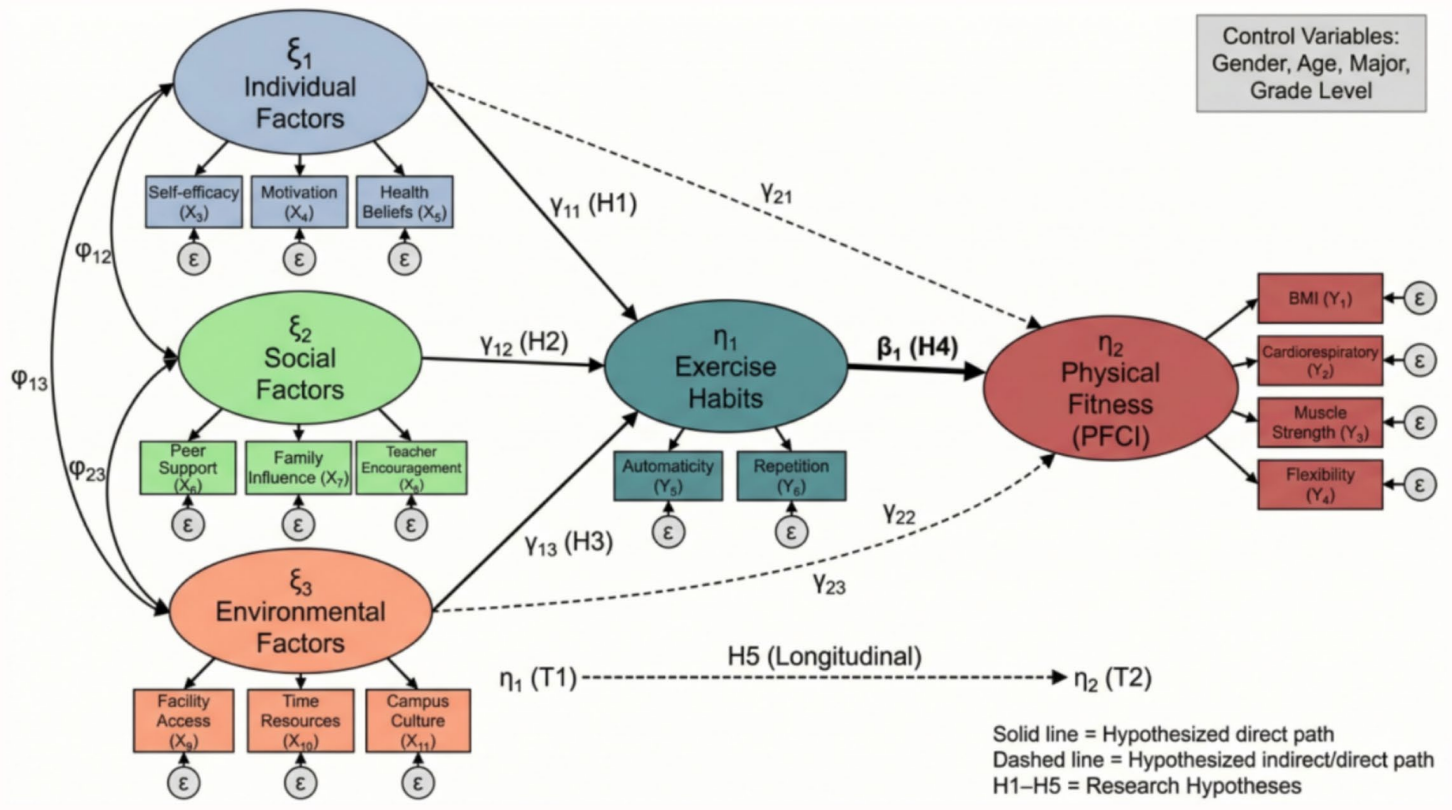
Research theoretical model diagram.

**Figure 3:**
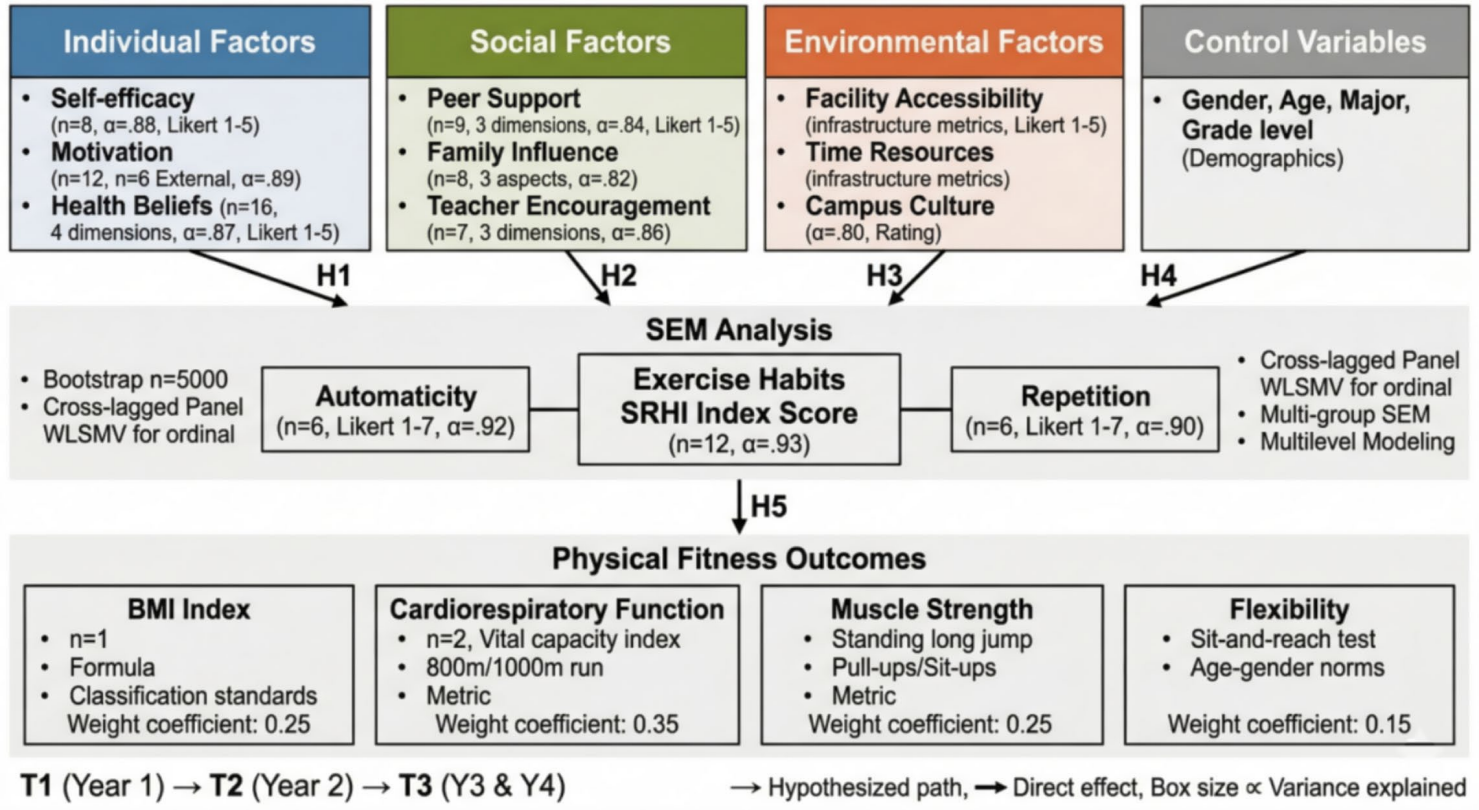
Research framework diagram.

The theoretical model (as shown in Figure 2) integrates core elements from the theory of planned behavior, social cognitive theory, and habit formation theory, constructing a comprehensive framework that includes antecedent variables, mediating variables, moderating variables, and outcome variables. The research framework diagram (as shown in Figure 3) further refines the operational definitions and measurement dimensions of each variable, laying the foundation for subsequent empirical analysis. Specifically, H1 through H3 address the direct and indirect pathways from multi-level factors to physical fitness outcomes, while H4 examines whether the mediating mechanism of exercise habits operates differently across subgroups defined by gender, grade level, and disciplinary background. H5 utilizes the longitudinal nature of the data to examine the temporal stability and predictive power of exercise habits on physical fitness trajectories over the four-year period.

## 3 Research design and methods

### 3.1 Research subjects and data collection

This study adopts a prospective longitudinal tracking design, selecting full-time undergraduate students enrolled in 2021 at a comprehensive university in northern China as research subjects. Through stratified random sampling, research samples were drawn from 12 colleges, covering different disciplinary categories such as science and engineering, liberal arts, medicine, and arts to ensure sample representativeness. After obtaining approval from the university ethics committee, the research team conducted a four-year tracking survey of these students from September 2021 to June 2025. Data collection work was divided into three main stages: physical fitness and health test data collection in the fall semester of each academic year, collection of information on exercise habits, psychological factors, and environmental factors through questionnaire surveys in the spring semester, and supplementation of qualitative data through in-depth interviews and focus group discussions during the summer. Of the 1,286 students initially recruited, 1,154 completed the full four-year tracking survey, with an effective tracking rate of 89.74%. The loss of samples was mainly due to transfer, leave of absence, or personal reasons for withdrawing from the study. Propensity score matching analysis showed no significant differences in baseline characteristics between lost samples and retained samples, indicating that sample loss did not produce systematic bias in research results.

**Figure 4:**
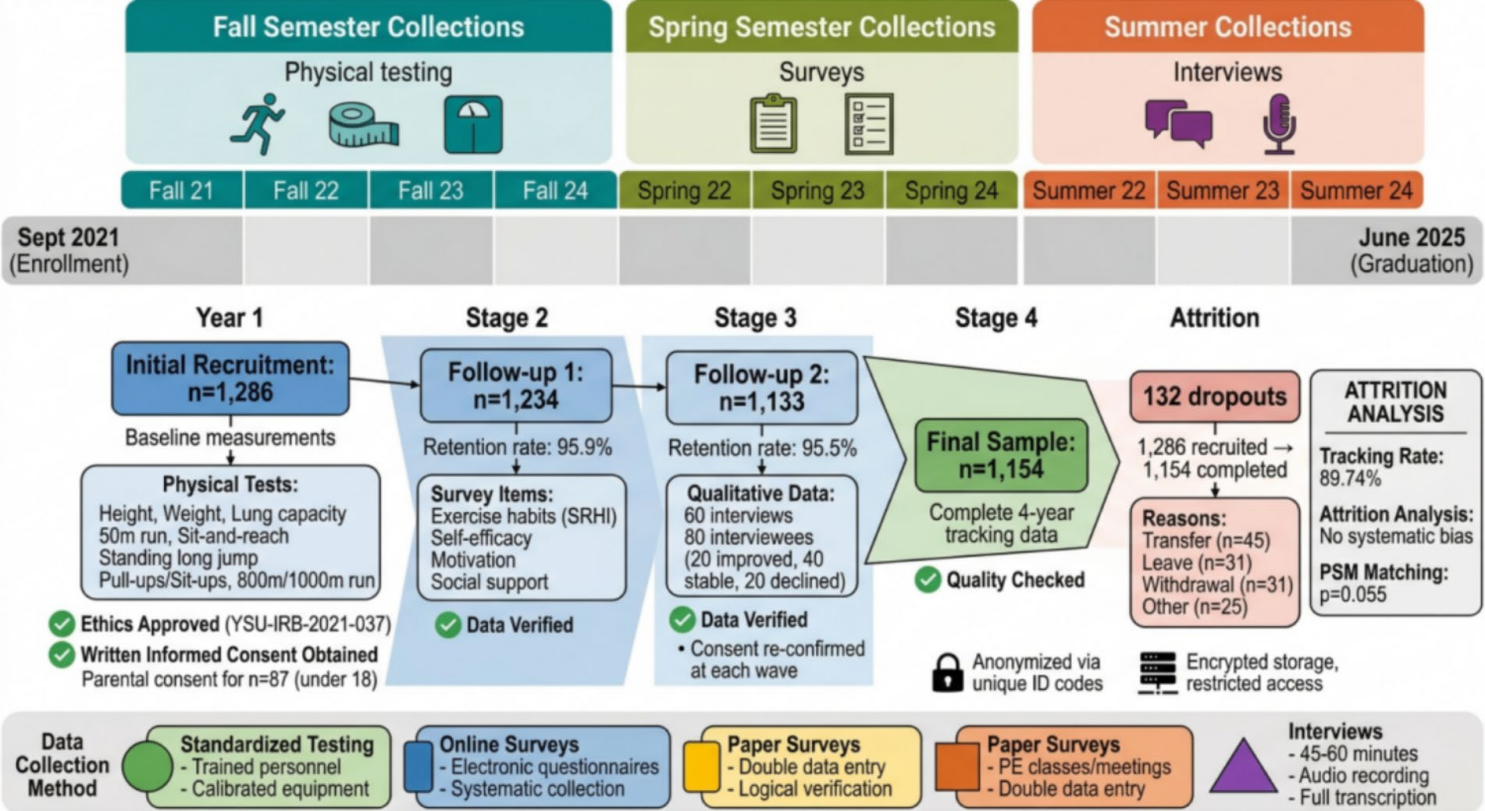
Data collection process and timeline diagram.

As shown in Figure 4, the data collection process strictly followed standardized procedures. Physical fitness and health tests were completed by professionally trained and certified testers using calibrated instruments and equipment in a standardized testing environment. Test items included height and weight measurement, vital capacity test, 50-meter run, sit-and-reach, standing long jump, pull-ups (for males) or sit-ups (for females), 800-meter run (for females) or 1,000-meter run (for males), and other items specified in the National Student Physical Fitness and Health Standards. Each test was measured twice and the best result was recorded. Questionnaire surveys were conducted using a combination of online and paper methods, with electronic questionnaire links pushed through the student affairs management system. For students who did not complete online surveys in time, paper questionnaires were distributed during physical education classes or class meetings. All questionnaire data underwent double entry and logical verification to ensure data quality. In-depth interview subjects were selected through purposive sampling, including 20 typical cases each of significantly improved, stable, and significantly declined physical fitness and health levels. Interview content focused on themes such as the exercise habit formation process, perception of influencing factors, and experiences of health behavior change. Each interview lasted 45–60 min and was fully recorded and transcribed.

Regarding ethics approval and data transparency, this study was approved by the Institutional Review Board of Yanshan University (Approval No. YSU-IRB-2021-037, approved on June 15, 2021) and was conducted in accordance with the Declaration of Helsinki. Prior to participation, all students received a detailed written informed consent form explaining the study purpose, procedures, potential risks and benefits, confidentiality protections, and their right to withdraw at any time without consequences. Signed informed consent was obtained from all participants before baseline data collection. All personal data were anonymized through unique identification codes, and data files were stored on encrypted, password-protected servers with access restricted to authorized research team members. The de-identified dataset supporting the findings of this study is available from the corresponding author upon reasonable request. Interested researchers may contact the corresponding author to submit a data access application, which will be reviewed by the research ethics committee. A data sharing agreement specifying the purpose, scope, and confidentiality obligations will be required prior to data release.

### 3.2 Variable measurement and operational definition

The measurement of research variables adopted a combination of internationally used scales and self-designed questionnaires, with all measurement tools undergoing reliability and validity testing and cultural adaptation adjustment. Physical fitness and health outcomes, as dependent variables, were comprehensively evaluated through four dimensions. A composite Physical Fitness Composite Index (PFCI) was adopted as the primary outcome variable rather than individual fitness test items for three interconnected reasons. First, physical fitness is inherently a multidimensional construct; no single test item—whether BMI, cardiorespiratory endurance, muscular strength, or flexibility—can adequately capture an individual’s overall fitness status (33). Using separate test items as independent outcomes would fragment the analysis across multiple dependent variables, increasing the risk of Type I error from multiple comparisons while failing to represent the holistic nature of physical fitness. Second, the Chinese National Student Physical Fitness and Health Standards themselves employ a composite scoring system that integrates multiple fitness domains into a single overall score for official student fitness evaluation nationwide; the present composite index aligns with this established and widely recognized evaluation framework, enhancing the practical relevance of the findings. Third, because certain test items are gender-specific (pull-ups for males vs. sit-ups for females; 1,000-meter run for males vs. 800-meter run for females), direct comparison of raw scores across genders would be inappropriate. The composite index addresses this concern by first converting all raw scores into standardized scores based on age- and gender-specific national norms, thereby placing male and female performance on a comparable metric before aggregation. BMI index was calculated based on height and weight and classified according to Chinese adult standards. Cardiopulmonary function was expressed as the standardized score of vital capacity body weight index and middle-distance running performance. Muscle strength was evaluated by the percentile ranking of standing long jump, pull-ups, or sit-up performance. Flexibility was determined by comparing sit-and-reach test results with age and gender norms. Since certain test items are gender-specific (pull-ups for males vs. sit-ups for females; 1,000-meter run for males vs. 800-meter run for females), raw scores for each item were first converted into standardized scores based on the age- and gender-specific norms established in the National Student Physical Fitness and Health Standards. This standardization procedure ensured that male and female scores were placed on a comparable metric before being combined into the composite index, thereby addressing the inherent differences in test content across genders. After standardization of the four dimension scores, they were weighted and averaged to obtain the PFCI. The weight coefficients were determined through principal component analysis (PCA) conducted on the baseline (freshman year) standardized scores of the four fitness dimensions. The Kaiser-Meyer-Olkin (KMO) measure of sampling adequacy was 0.78, and Bartlett’s test of sphericity was significant (χ^2^ = 1,842.36, df = 6, *p* < 0.001), confirming the suitability of the data for PCA. The analysis extracted one principal component with an eigenvalue of 2.31, accounting for 57.75% of the total variance. The factor loadings on this component were 0.74 for BMI, 0.86 for cardiopulmonary function, 0.76 for muscle strength, and 0.59 for flexibility (see Table 1). Based on the relative magnitude of the squared factor loadings, the weight coefficients were determined as 0.25 for BMI, 0.35 for cardiopulmonary function, 0.25 for muscle strength, and 0.15 for flexibility.

**Table 1:** Principal component analysis results for physical fitness dimensions (*N* = 1,154).

Dimension	Factor loading	Squared loading	Normalized weight
BMI	0.74	0.548	0.25
Cardiopulmonary function	0.86	0.74	0.35
Muscle strength	0.76	0.578	0.25
Flexibility	0.59	0.348	0.15
Eigenvalue	2.31		
Variance explained (%)	57.75		
KMO	0.78		
Bartlett’s χ^2^	1,842.36*		

*p < 0.001; df = 6.

**Figure 5:**
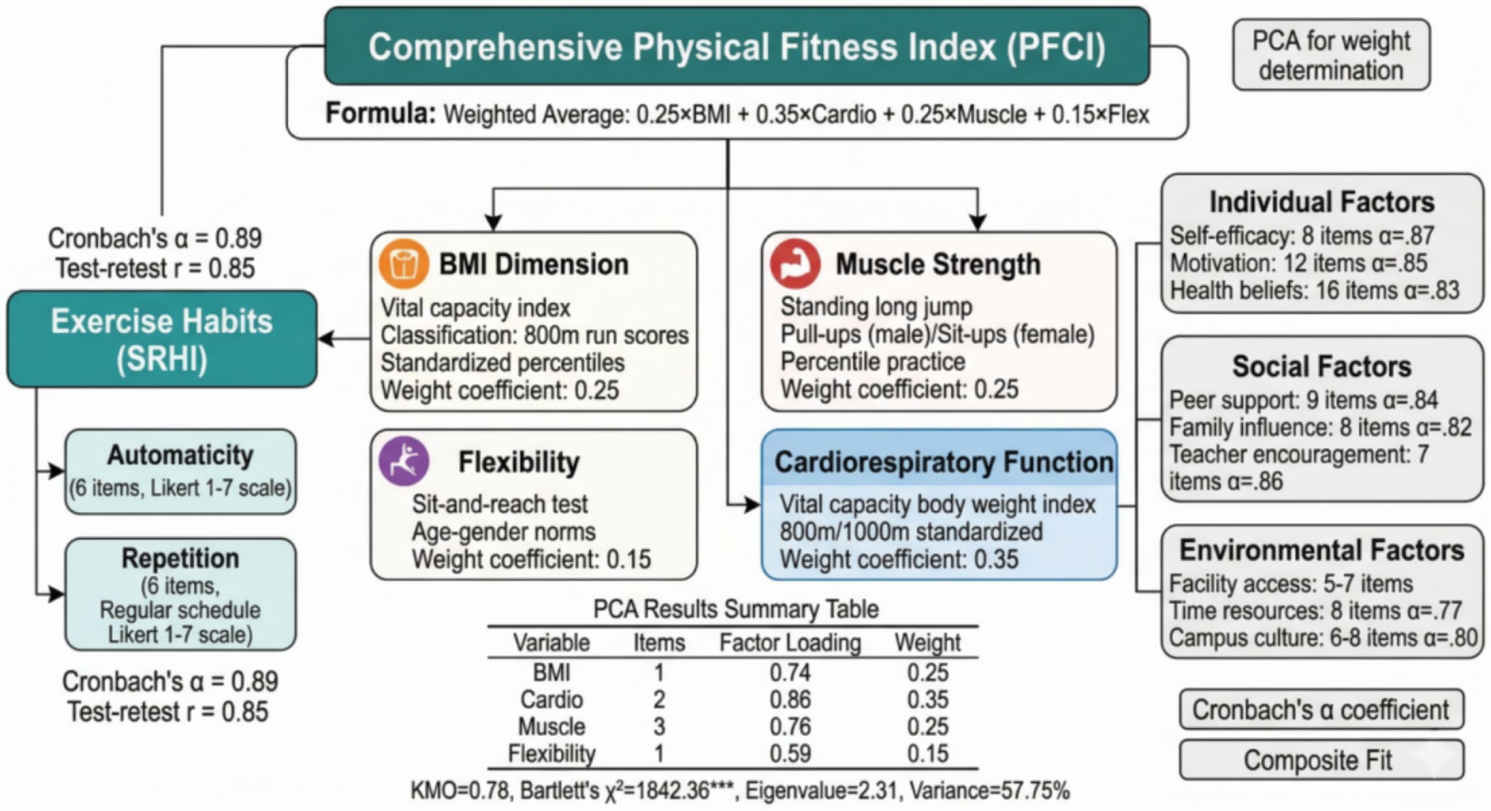
Variable measurement framework and dimensional composition diagram.

The assignment of the highest weight coefficient (0.35) to the cardiorespiratory dimension is supported not only by its empirical dominance in the factor structure but also by well-established evidence from health science and exercise physiology. Cardiorespiratory fitness is widely recognized as the single strongest physiological predictor of all-cause mortality and cardiovascular disease risk across the lifespan (34, 35). A meta-analysis of 33 prospective studies encompassing over 100,000 participants demonstrated that each one-MET increase in cardiorespiratory fitness was associated with a 13% reduction in all-cause mortality and a 15% reduction in cardiovascular mortality (34). The physiological basis for this primacy lies in the integrative nature of cardiorespiratory function: maximal oxygen uptake (VO₂max) reflects the coordinated performance of the pulmonary system (gas exchange efficiency), the cardiovascular system (cardiac output and peripheral circulation), and the musculoskeletal system (oxygen extraction and utilization), thereby serving as a comprehensive indicator of overall physiological capacity (35). Moreover, among college-aged populations specifically, longitudinal evidence indicates that cardiorespiratory fitness established during young adulthood is a significant predictor of metabolic health and chronic disease risk in later life (36). By contrast, while muscular strength, flexibility, and body composition each contribute meaningfully to health outcomes, their individual predictive power for long-term mortality and morbidity is comparatively weaker than that of cardiorespiratory endurance (37). These empirically derived weights are also broadly consistent with the emphasis placed on cardiorespiratory endurance in the National Student Physical Fitness and Health Standards, which assigns the greatest scoring proportion to middle- and long-distance running performance among all test items, thereby providing convergent support from both physiological evidence and policy frameworks for the validity of the composite index. Figure 5 shows the variable measurement framework, displaying the specific composition and calculation methods of each dimension indicator.

Exercise habits, as the core mediating variable, were measured using the Self-Report Habit Index scale. The scale includes 12 items across two sub-dimensions: automaticity and repetition. The automaticity dimension measures the spontaneity of exercise behavior with 6 items such as “I start exercising unconsciously” and “Exercise is an instinctive response for me.” The repetition dimension assesses the regularity and persistence of exercise behavior with 6 items such as “I engage in physical exercise at fixed times every week” and “Even when encountering difficulties, I stick to my exercise plan.” A Likert seven-point scale was used, with scores from “strongly disagree” to “strongly agree” assigned values of 1–7. The scores of the two dimensions were added and averaged as an indicator of exercise habit intensity. The Cronbach’s *α* coefficient of the scale in this study was 0.89, and the test–retest reliability was 0.85.

The measurement of multiple influencing factors covered three levels: individual, social, and environmental, totaling 28 observed variables. Individual-level factors included exercise self-efficacy measured by the Exercise Confidence Scale with 8 items such as “I believe I can overcome difficulties in exercise.” Exercise motivation was assessed through the internal motivation and external motivation subscales of the Exercise Motivation Scale, each with 6 items. Health beliefs were measured using the Health Belief Model Scale, including four dimensions: perceived threat, perceived benefits, perceived barriers, and action cues, totaling 16 items. Social-level factors involved peer support measured through three dimensions of the Social Support Scale: emotional support, informational support, and instrumental support, totaling 9 items. Family influence was assessed using the Family Exercise Atmosphere Questionnaire, including three aspects: parental role modeling, family encouragement, and joint participation, totaling 8 items. Teacher encouragement was measured through the Physical Education Teacher Support Perception Scale, including three dimensions: skill guidance, emotional motivation, and personalized attention, totaling 7 items.

Given that multiple core variables (exercise habits, self-efficacy, exercise motivation, peer support, etc.) were measured through self-report questionnaires within the same survey, the risk of common method bias was assessed using Harman’s single-factor test (38). All items from the self-report measures were entered into an unrotated exploratory factor analysis. The results indicated that the first factor accounted for 23.67% of the total variance, which is well below the commonly used threshold of 40%. Additionally, a confirmatory factor analysis was conducted comparing a single-factor model (in which all items loaded onto one latent factor) with the theoretically specified multi-factor measurement model. The single-factor model showed poor fit (χ^2^/df = 8.42, CFI = 0.624, TLI = 0.598, RMSEA = 0.112), whereas the multi-factor model demonstrated acceptable fit (χ^2^/df = 2.18, CFI = 0.951, TLI = 0.943, RMSEA = 0.045). The substantial difference in model fit between the two models (Δχ^2^ = 3,847.25, Δdf = 15, *p* < 0.001) further confirmed that common method bias did not pose a serious threat to the validity of the present findings.

### 3.3 Empirical model construction

Based on the theoretical framework and research hypotheses, this study constructed a multi-level structural equation model to test the mediating mechanism of exercise habits in the process through which multiple factors predict physical fitness and health. Before describing the specific model specifications, it is important to clarify the overall analytical strategy, which was organized into a primary analysis and two tiers of supporting analyses, each serving a clearly defined and distinct purpose.

The primary analysis consisted of structural equation modeling (SEM), which tested the hypothesized mediation model and estimated the direct and indirect effects of individual, social, and environmental factors on physical fitness through exercise habits (addressing H1, H2, and H4). This constitutes the central statistical evidence for the paper’s core argument regarding the mediating role of exercise habits. All subsequent analyses serve to extend, contextualize, or verify these primary mediation findings rather than to replace them.

The first tier of supporting analyses employed two complementary longitudinal methods. Latent growth curve modeling was used to characterize the trajectories of change in exercise habits and physical fitness over the four-year period, while cross-lagged panel analysis examined the temporal sequence and direction of predictive relationships between exercise habits and physical fitness (addressing H5). Additionally, moderating effects of gender, grade level, and disciplinary background were tested through multi-group SEM and interaction term analysis (addressing H3 and H4). These analyses provide important longitudinal and contextual evidence that extends the primary mediation findings by examining temporal dynamics and subgroup variations.

The second tier of supporting analyses consisted of robustness checks. Instrumental variable estimation and propensity score matching were conducted as supplementary methods to address potential endogeneity concerns and verify the stability of the main SEM findings under alternative analytical assumptions. These supplementary analyses are reported to demonstrate that the primary results are not artifacts of specific methodological choices, but they do not constitute the main evidence base of the study.

**Figure 6:**
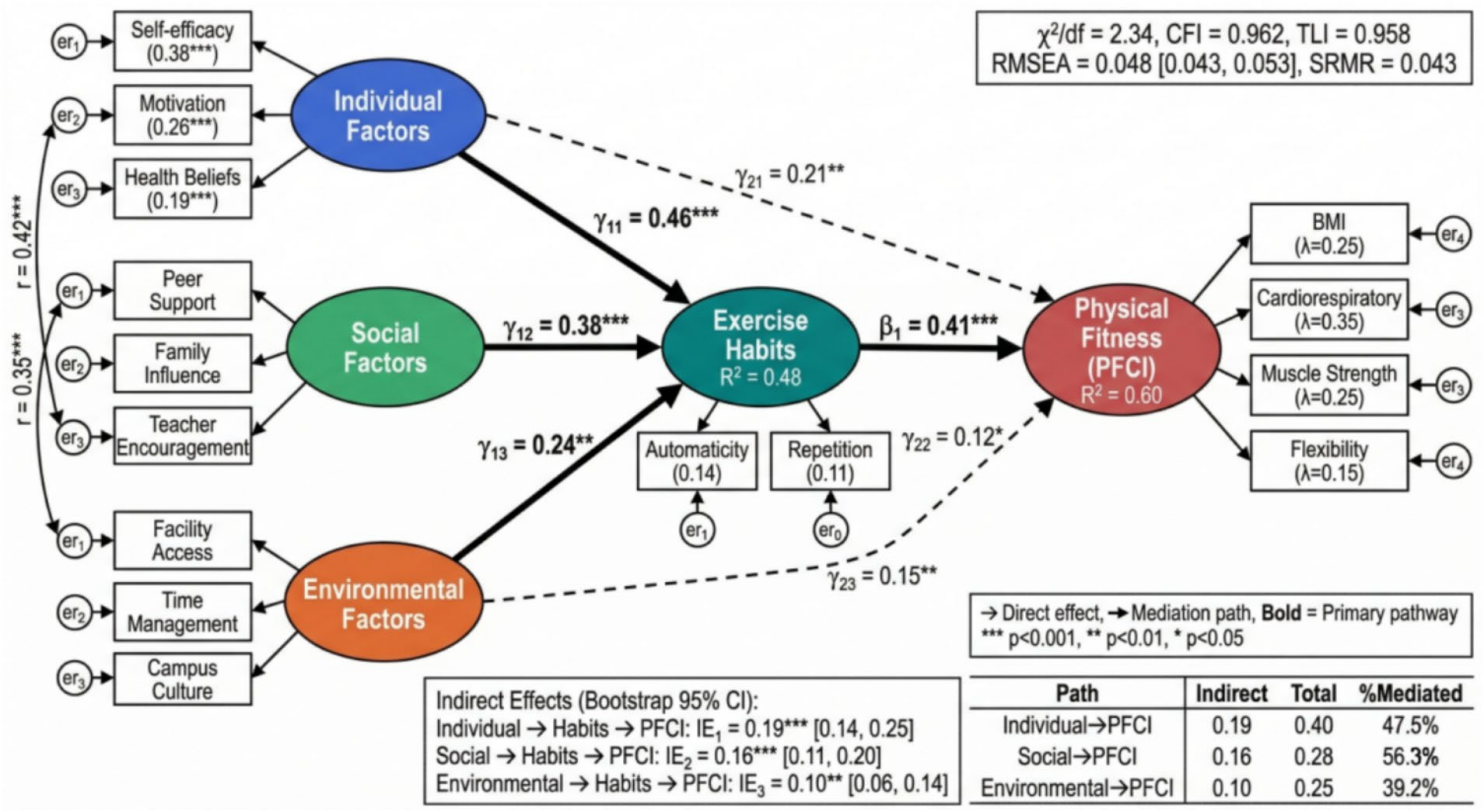
Structural equation model path diagram.

This hierarchical organization ensures that readers can clearly distinguish between the core evidence supporting the paper’s central argument (SEM-based mediation analysis) and the ancillary analyses that strengthen, extend, or verify those findings. Model setting followed latent variable modeling principles and considered the nested structure characteristics of time series data. As shown in Figure 6, the measurement model part established the correspondence between latent variables and observation indicators. The physical fitness and health latent variable $\eta_{1}$ was reflected by four observed variables: BMI index $\left(Y_{1}\right)$ , cardiopulmonary function $\left(Y_{2}\right)$ , muscle strength $\left(Y_{3}\right)$ , and flexibility $\left(Y_{4}\right)$ . Its measurement equation is expressed as Equation 1:


$$Y_{i}=\lambda_{i}^{y}\eta_{1}+\varepsilon_{i}\;(i=1,2,3,4)$$ (1)

Where $\lambda_{i}^{y}$ is the factor loading, indicating the loading coefficient of observed variable $Y_{i}$ on latent variable $\eta_{1}$ ; $\varepsilon_{i}$ is the measurement error term, assumed to follow a normal distribution with mean 0 and variance $\theta_{i}$ and to be independent of the latent variable.

The exercise habit latent variable $\xi_{1}$ was measured by two observed variables: automaticity score $\left(X_{1}\right)$ and repetition score $\left(X_{2}\right)$ . The individual factors latent variable *ξ*₂ was reflected by three observed variables: exercise self-efficacy $\left(X_{3}\right)$ , exercise motivation $\left(X_{4}\right)$ , and health beliefs $\left(X_{5}\right)$ . The social factors latent variable ξ₃ consisted of peer support $\left(X_{6}\right)$ , family influence $\left(X_{7}\right)$ , and teacher encouragement $\left(X_{8}\right)$ . The environmental factors latent variable $\xi_{4}$ included three indicators: facility accessibility $\left(X_{9}\right)$ , time resources $\left(X_{10}\right)$ , and campus culture $\left(X_{11}\right)$ . The corresponding measurement equation is Equation 2:


$$X_{j}=\lambda_{j}^{x}\xi_{k}+\delta_{j}\;\;(j=1,\ldots ,11;k=1,\ldots ,4)$$ (2)

Where $\lambda_{j}^{x}$ is the factor loading of exogenous observed variable $X_{j}$ on the corresponding latent variable $\xi_{k}$ ; $\delta_{j}$ is the measurement error, assumed to follow an independent identical distribution and be uncorrelated with the latent variable.

The structural model part describes the predictive relationship paths between latent variables. Considering the mediating role of exercise habits, it was set that individual factors, social factors, and environmental factors not only have a direct predictive relationship with exercise habits but also indirectly predict physical fitness and health outcomes through exercise habits. The structural equation is expressed as Equations 3 and 4:


$$\xi_{1}=\gamma_{11}\xi_{2}+\gamma_{12}\xi_{3}+\gamma_{13}\xi_{4}+\zeta_{1}$$ (3)

$$\eta_{1}=\beta_{11}\xi_{1}+\gamma_{21}\xi_{2}+\gamma_{22}\xi_{3}+\gamma_{23}\xi_{4}+\zeta_{2}$$ (4)

Where $\gamma_{11}$ , $\gamma_{12}$ , $\gamma_{13}$ represent the path coefficients from individual factors, social factors, and environmental factors to exercise habits, respectively; *β*₁₁ represents the path coefficient from exercise habits to physical fitness and health; *γ*₂₁, γ₂₂, γ₂₃ represent the direct paths from the three types of factors to physical fitness and health, respectively; *ζ*₁ and ζ₂ are the residual terms of the structural equation.

To test the significance and effect size of the mediating effect, the indirect effects generated by each factor through exercise habits were calculated. The indirect effect of individual factors $IE_{1}$ , the indirect effect of social factors $IE_{2}$ , and the indirect effect of environmental factors $IE_{3}$ are, respectively, expressed as Equation 5:


$$\begin{array}{l} IE_{1}=\gamma_{11}\times \beta_{11}\\ IE_{2}=\gamma_{12}\times \beta_{11}\\ IE_{3}=\gamma_{13}\times \beta_{11} \end{array}$$ (5)

The total effect TE equals the sum of the direct effect DE and indirect effect IE . The calculation formula for the proportion of mediating effect PM is Equation 6:


$$\mathrm{PM}=\frac{\mathrm{IE}}{\mathrm{TE}}=\frac{\mathrm{IE}}{\mathrm{DE}+\mathrm{IE}}$$ (6)

Considering the dynamic characteristics of longitudinal data and individual heterogeneity, a cross-lagged panel model was further constructed to identify the direction and temporal sequence of predictive relationships between variables. It was set that physical fitness and health levels at time t are not only predicted by current exercise habits but also by previous physical fitness and health levels and exercise habits. The dynamic panel model is expressed as Equation 7:


$$\mathrm{PH}F_{\mathrm{it}}=\mathrm{\rho PH}F_{i,t-1}+\beta_{1}EH_{\mathrm{it}}+\beta_{2}EH_{i,t-1}+\alpha_{i}+\mu_{\mathrm{it}}$$ (7)

Where $\mathrm{PH}F_{\mathrm{it}}\;$ represents individual i’s physical fitness and health level at time t; EH_it is the current exercise habit intensity; *ρ* is the autoregressive coefficient of physical fitness and health, reflecting the persistence of health status; β₁ and β₂ are the predictive coefficients of current and lagged exercise habits, respectively; *α*ᵢ is the unobserved individual fixed effect, controlling for time-invariant individual characteristics; $\mu_{\mathrm{it}}$ is the random disturbance term.

To address potential endogeneity issues and reverse causality, instrumental variable methods were used for robustness testing. Parental exercise frequency and community sports facility density were selected as instrumental variables for exercise habits. The first-stage regression and second-stage regression of instrumental variable estimation are respectively Equations 8 and 9:


$$EH_{\mathrm{it}}=\pi_{0}+\pi_{1}Z_{1\mathrm{it}}+\pi_{2}Z_{2\mathrm{it}}+\pi_{3}X_{\mathrm{it}}+v_{\mathrm{it}}\;$$ (8)

$$\mathrm{PH}F_{\mathrm{it}}=\theta_{0}+\theta_{1}\hat{\mathrm{EH}}\mathrm{it}+\theta_{2}\mathrm{Xit}+e_{\mathrm{it}}$$ (9)

Where $Z_{1\mathrm{it}}$ and $Z_{2\mathrm{it}}$ are the two instrumental variables: parental exercise frequency and community facility density, respectively; $X_{\mathrm{it}}$ is the control variable vector; π and θ are parameters to be estimated; v and e are error terms; ${\hat{\mathrm{EH}}}_{\mathrm{it}}$ is the predicted value of exercise habits obtained from the first stage.

### 3.4 Analysis methods and tools

Data analysis adopted the analytical strategy outlined in Section 3.3—comprising a primary SEM-based mediation analysis and two tiers of supporting analyses—implemented through a combined analysis approach of descriptive statistics, correlation analysis, structural equation modeling, and multilevel modeling. SPSS 26.0 was used for data cleaning, descriptive statistics, common method bias testing, and preliminary correlation analysis. Mplus 8.3 was used for structural equation model estimation, confirmatory factor analysis, mediation effect testing, latent growth curve modeling, and multi-group invariance testing. Stata 17.0 was used to complete dynamic panel model and instrumental variable regression analysis. The significance level for all statistical tests was set at α = 0.05. Model estimation used the maximum likelihood estimation method to handle continuous variables and the weighted least squares method for ordinal categorical variables. Missing data were handled through full information maximum likelihood estimation to fully utilize all available information. Model fit was comprehensively evaluated through multiple indicators such as chi-square test, Comparative Fit Index (CFI), Tucker-Lewis Index (TLI), Root Mean Square Error of Approximation (RMSEA), and Standardized Root Mean Square Residual (SRMR). Good fit criteria were CFI > 0.95, TLI > 0.95, RMSEA < 0.06, SRMR < 0.08.

**Figure 7:**
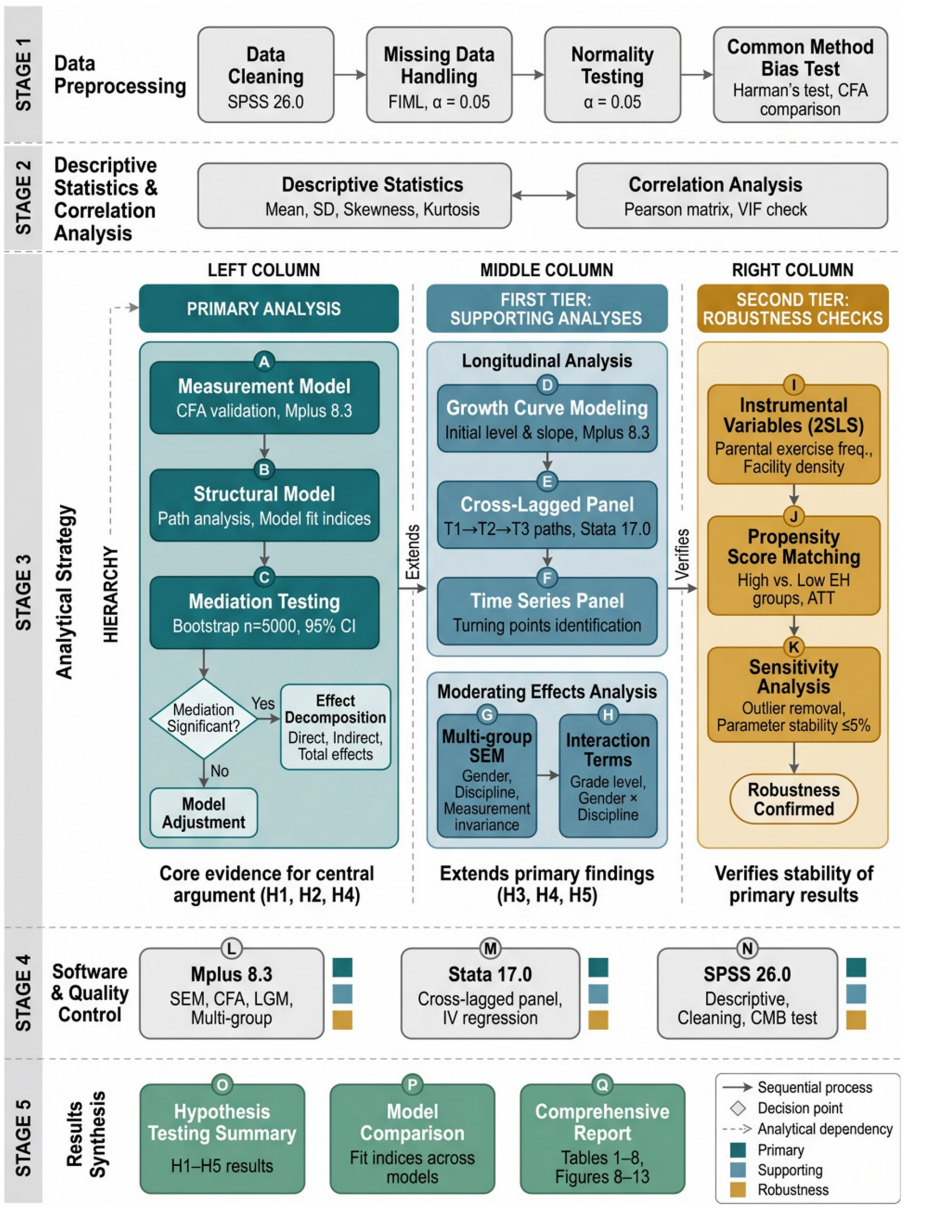
Data analysis technical route diagram.

Mediation effect testing used the Bootstrap method with 5,000 resampling iterations to construct 95% confidence intervals. If the confidence interval did not contain 0, the mediation effect was judged to be significant. Sobel test statistics were also calculated as supplementary verification. Moderating effects were tested by adding interaction terms to the structural equation, using mean centering to reduce multicollinearity problems. Specifically, the moderating effects of gender, disciplinary background, and grade level were examined. For gender and disciplinary background, multi-group SEM was conducted to compare path coefficients across subgroups, with measurement invariance tested sequentially (configural, metric, and scalar). For grade level, time-varying interaction terms were introduced into the cross-lagged panel model to examine whether the predictive relationships between exercise habits and physical fitness varied across different academic years. Additionally, the two-way interaction of gender × disciplinary background on the path from time management pressure to exercise habits was tested by including the multiplicative interaction term in the structural model. Figure 7 shows the analysis process, displaying the complete technical route from data preprocessing to model testing. The latent growth curve model results are reported with complete model fit indicators (χ^2^/df, CFI, TLI, RMSEA, SRMR), group-specific growth parameters, and formal inter-group slope difference tests to ensure full transparency of the longitudinal findings. Multilevel model analysis considered the nested structure of data, with students nested within classes and classes nested within colleges. A three-level structure was estimated through random intercept models and random slope models. The intraclass correlation coefficient (ICC) was used to evaluate the relative contribution of variance components at different levels. Model comparison was conducted through likelihood ratio tests and information criteria.

## 4 Empirical results analysis

### 4.1 Descriptive statistics and correlation analysis

The basic characteristic analysis of the research sample showed that among the 1,154 college students who completed the four-year tracking, males accounted for 52.3% (604 people), females accounted for 47.7% (550 people), the age range at enrollment was 17–20 years, with an average age of 18.42 years (SD = 0.68). In terms of disciplinary distribution, science and engineering students accounted for 38.7%, liberal arts students accounted for 29.5%, medical students accounted for 18.4%, and arts students accounted for 13.4%. This distribution was basically consistent with the overall student structure of the school. Table 2 presents descriptive statistical results of major research variables at four time points. The PFCI decreased year by year from 73.26 points (SD = 8.94) in the first year to 68.52 points (SD = 10.37) in the fourth year, with a decrease of 6.47%. This downward trend was evident in students of different genders and disciplinary backgrounds, but there were differences in degree.

**Table 2:** Descriptive statistics of main variables over 4 years (*N* = 1,154).

Variable	Freshman year *M*	Freshman year SD	Sophomore year *M*	Sophomore year SD	Junior year *M*	Junior year SD	Senior year *M*	Senior year SD
PFCI	73.26	8.94	71.83	9.27	70.35	9.68	68.52	10.37
BMI	21.48	3.12	21.96	3.34	22.28	3.45	22.84	3.58
Cardiopulmonary function	74.35	11.26	72.14	11.89	69.47	12.56	67.23	13.18
Muscle strength	71.89	9.87	70.56	10.23	68.23	10.89	66.45	11.34
Flexibility	73.56	8.45	72.89	8.73	71.56	9.12	70.23	9.48
Exercise habit intensity	4.82	1.34	4.35	1.47	3.89	1.56	3.54	1.69
Exercise self-efficacy	5.14	1.08	4.87	1.15	4.56	1.23	4.28	1.34
Peer support	5.26	0.97	4.93	1.04	4.67	1.12	4.45	1.21
Facility accessibility	5.78	0.86	5.72	0.89	5.65	0.92	5.58	0.95

PFCI = Physical Fitness Composite Index. Exercise Habit Intensity, Exercise Self-efficacy, Peer Support, and Facility Accessibility are measured on Likert scales (1–7). BMI is in kg/m^2^. Cardiopulmonary Function, Muscle Strength, and Flexibility are standardized scores (0–100).

**Figure 8:**
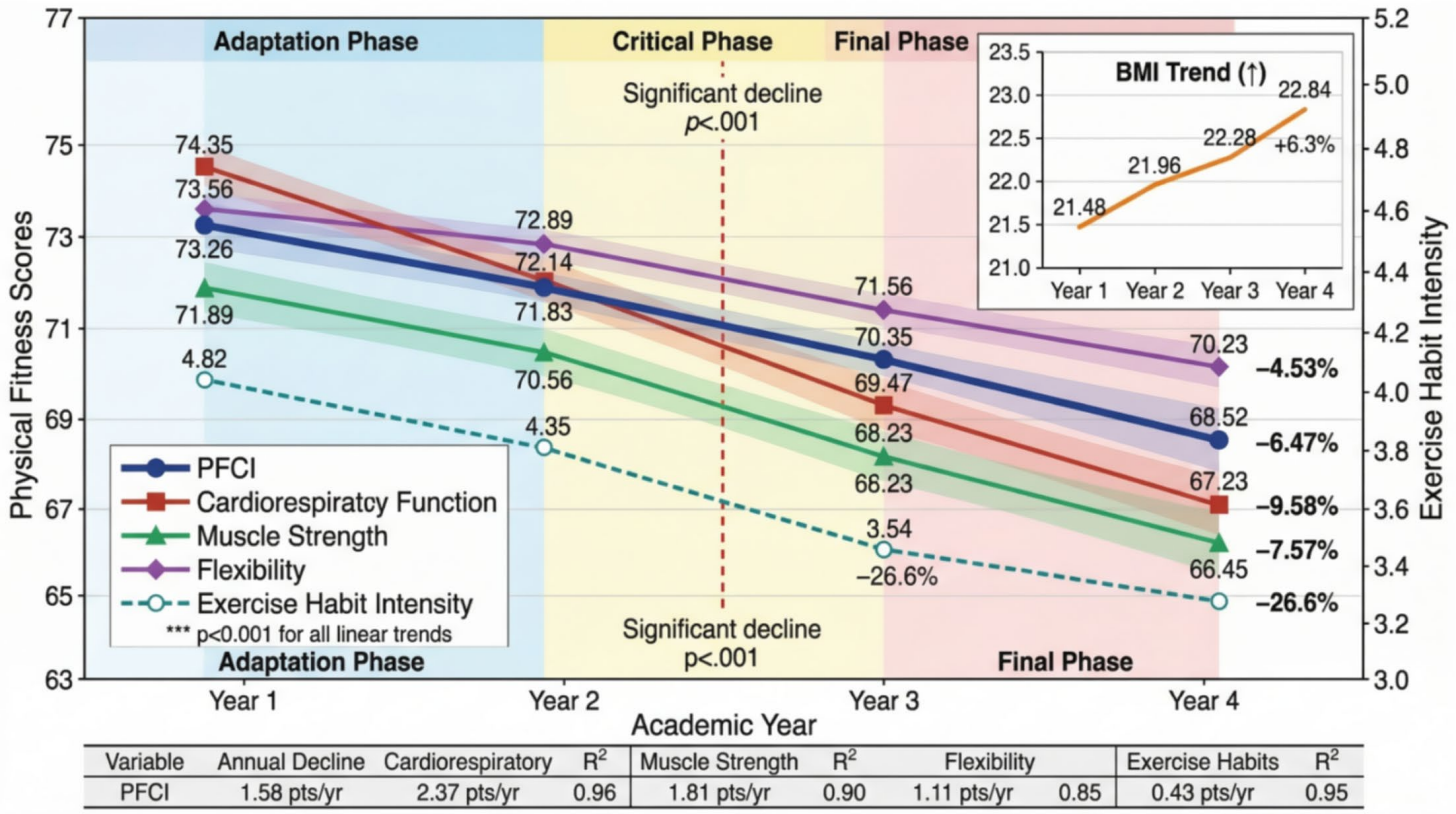
Four-year change trajectory diagram of main variables.

Exercise habit intensity showed a more obvious downward trend, continuously decreasing from 4.82 (SD = 1.34) in the first year to 3.54 (SD = 1.69) in the fourth year, with a decrease of 26.6%. The decrease in the automaticity dimension (31.2%) was significantly greater than that in the repetition dimension (22.8%), indicating that students’ exercise behavior gradually transformed from spontaneous activities to states requiring willpower to maintain. As shown in Figure 8, the change trajectory diagram clearly displays the dynamic change patterns of different dimensional indicators. The decline rate of cardiopulmonary function was most significant during the sophomore to junior year period, while BMI growth showed a relatively stable linear trend.

Correlation analysis results revealed association patterns between variables. Table 3 presents a Pearson correlation coefficient matrix of key variables. Exercise habit intensity showed a significant positive correlation with the PFCI at all four time points, with correlation coefficients gradually strengthening from 0.42 in the first year to 0.58 in the fourth year (*p* < 0.001). This strengthening correlation suggests that the predictive relevance of exercise habits for physical fitness and health increases over time. The correlation between exercise self-efficacy and exercise habits remained at a moderate to high level of 0.51–0.56. The correlation coefficient between peer support and exercise habits ranged from 0.43–0.49. Among environmental factors, the correlation of facility accessibility was relatively weak but still significant (*r* = 0.28–0.35, *p* < 0.01).

**Table 3:** Correlation coefficient matrix of main variables.

Variable	1	2	3	4	5	6	7	8
1. PFCI	1							
2. Exercise habit intensity	0.52***	1						
3. Exercise self-efficacy	0.46***	0.54***	1					
4. Exercise motivation	0.38***	0.47***	0.62***	1				
5. Peer support	0.35***	0.46***	0.43***	0.39***	1			
6. Family influence	0.29***	0.34***	0.36***	0.32***	0.41***	1		
7. Facility accessibility	0.24**	0.31***	0.28***	0.25**	0.23**	0.19*	1	
8. Time management pressure	−0.41***	−0.48***	−0.39***	−0.35***	−0.27***	−0.21**	−0.18*	1

*p < 0.05, **p < 0.01, *p < 0.001. Values represent Pearson correlation coefficients aggregated across four measurement waves. PFCI = Physical Fitness Composite Index.

### 4.2 Multiple regression analysis results

Hierarchical regression analysis was used to test the predictive effects of different categories of factors on physical fitness and health and their relative contributions. Model 1 only included control variables (gender, age, disciplinary background). Model 2 added individual-level factors. Model 3 further included social-level factors. Model 4 included environmental-level factors. Model 5 finally added exercise habit variables to examine whether the inclusion of exercise habits reduced the strength of other predictors, which would be consistent with a potential mediating role. The results in Table 4 show that the addition of individual factors increased the model’s explanatory power from 8.7 to 31.4% (Δ*R*^2^ = 0.227, *p* < 0.001). Among them, the standardized regression coefficient of exercise self-efficacy was 0.38 (*p* < 0.001), making it the strongest predictor among individual factors. The coefficients of exercise motivation and health beliefs were 0.26 and 0.19, respectively, both reaching statistical significance.

**Table 4:** Hierarchical regression analysis results of factors predicting PFCI.

Factor category	Predictor variable	Model 1	Model 2	Model 3	Model 4	Model 5
Control variables	Gender (Male = 1)	0.15***	0.09*	0.08*	0.07	0.05
	Age	−0.06	−0.04	−0.03	−0.02	−0.01
	Disciplinary Background	0.08*	0.05	0.04	0.03	0.02
Individual factors	Exercise self-efficacy	–	0.38***	0.29***	0.24***	0.15**
	Exercise motivation	–	0.26***	0.21***	0.18**	0.11*
	Health beliefs	–	0.19***	0.16**	0.14**	0.09
Social factors	Peer support	–	–	0.23***	0.19***	0.10*
	Family influence	–	–	0.17**	0.15**	0.08
	Teacher encouragement	–	–	0.14**	0.12*	0.06
Environmental factors	Facility accessibility	–	–	–	0.13**	0.07
	Time management	–	–	–	−0.21***	−0.12*
	Campus culture	–	–	–	0.11*	0.05
	Exercise habit intensity	–	–	–	–	0.42***
*R* ^2^		0.087	0.314	0.427	0.486	0.598
Δ*R*^2^		–	0.227***	0.113***	0.059***	0.112***
*F-*value		36.52***	87.94***	106.28***	113.67***	148.93***

Values in the table are standardized regression coefficients β; *p < 0.05, **p < 0.01, *p < 0.001. PFCI = Physical Fitness Composite Index.

The inclusion of social factors further enhanced the model’s explanatory power (Δ*R*^2^ = 0.113, *p* < 0.001). Peer support emerged as the most important predictor among social factors (*β* = 0.23, *p* < 0.001), while the contributions of family influence and teacher encouragement were relatively small but still significant. The contribution of environmental factors was relatively limited (Δ*R*^2^ = 0.059, *p* < 0.001). Time management pressure showed a significant negative predictive effect (*β* = −0.21, *p* < 0.001), consistent with the potential crowding-out effect of academic burden on health behavior. When the exercise habit variable was added to the model, the explanatory power increased significantly to 59.8% (Δ*R*^2^ = 0.112, *p* < 0.001). The regression coefficient of exercise habits reached 0.42 (*p* < 0.001), while the coefficients of other predictor variables decreased notably, a pattern that is consistent with the hypothesis that exercise habits may serve as a mediator in the relationships between the multi-level factors and physical fitness outcomes. However, it should be noted that the attenuation of predictor coefficients upon inclusion of a potential mediator does not, by itself, establish a causal mediation mechanism; rather, it provides preliminary statistical evidence that warrants further examination through formal mediation analysis.

### 4.3 Mediation effect testing

**Figure 9:**
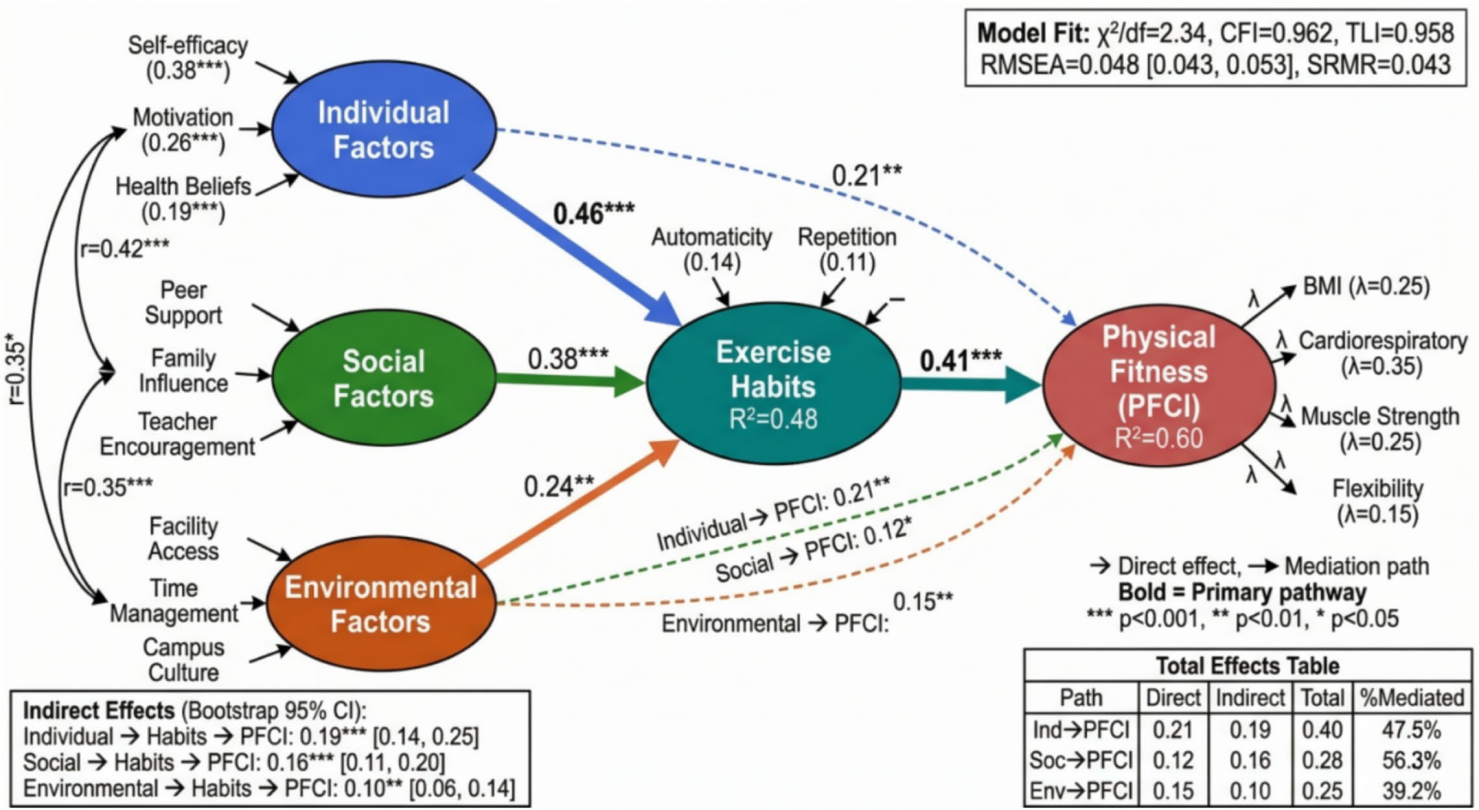
Structural equation model standardized path coefficient diagram.

Structural equation model analysis further examined the potential mediating mechanism of exercise habits in the process through which multiple factors are associated with physical fitness and health. Model fit indicators showed that the data had good compatibility with the theoretical model (χ^2^/df = 2.34, CFI = 0.962, TLI = 0.958, RMSEA = 0.048, SRMR = 0.043). As shown in Figure 9, the path coefficient diagram displays standardized path coefficients between latent variables. The path from individual factors to exercise habits was 0.46 (*p* < 0.001), the path to physical fitness and health was 0.21 (*p* < 0.01), and the indirect effect generated through exercise habits was 0.19 (95% CI: 0.14–0.25). The proportion of the indirect effect to total effect was 47.5%, indicating that exercise habits functioned as a statistically significant partial mediator in the predictive pathway from individual factors to physical fitness and health.

The path of social factors predicting physical fitness and health through exercise habits was also examined. The path coefficient from social factors to exercise habits was 0.38 (*p* < 0.001), and the path coefficient from exercise habits to physical fitness and health was 0.41 (*p* < 0.001). The calculated indirect effect was 0.156 (95% CI: 0.11–0.20), accounting for 56.3% of the total effect. The predictive path of environmental factors was relatively complex. Its path coefficient to exercise habits was relatively weak (*β* = 0.24, *p* < 0.01), but it produced a significant moderating effect through interaction with individual factors and social factors. Table 5 presents detailed effect decomposition results for different paths, including estimates of direct effects, indirect effects, and total effects and their confidence intervals.

**Table 5:** Mediation effect decomposition and Bootstrap test results.

Path	Direct effect	Indirect effect	Total effect	Mediation effect proportion	95% CI
Individual factors→PFCI	0.21**	0.19***	0.40***	47.50%	[0.14, 0.25]
Social factors→PFCI	0.12*	0.16***	0.28***	56.30%	[0.11, 0.20]
Environmental factors→PFCI	0.15**	0.10**	0.25***	39.20%	[0.06, 0.14]
Exercise self-efficacy→PFCI	0.18**	0.22***	0.40***	55.00%	[0.17, 0.28]
Peer support→PFCI	0.09	0.14***	0.23***	60.90%	[0.10, 0.19]
Time management→PFCI	−0.16**	−0.12**	−0.28***	42.90%	[−0.17, −0.08]

Based on 5,000 Bootstrap samples; *p < 0.05, **p < 0.01, *p < 0.001. PFCI = Physical Fitness Composite Index. All indirect effects are through exercise habits as the mediator.

**Figure 10:**
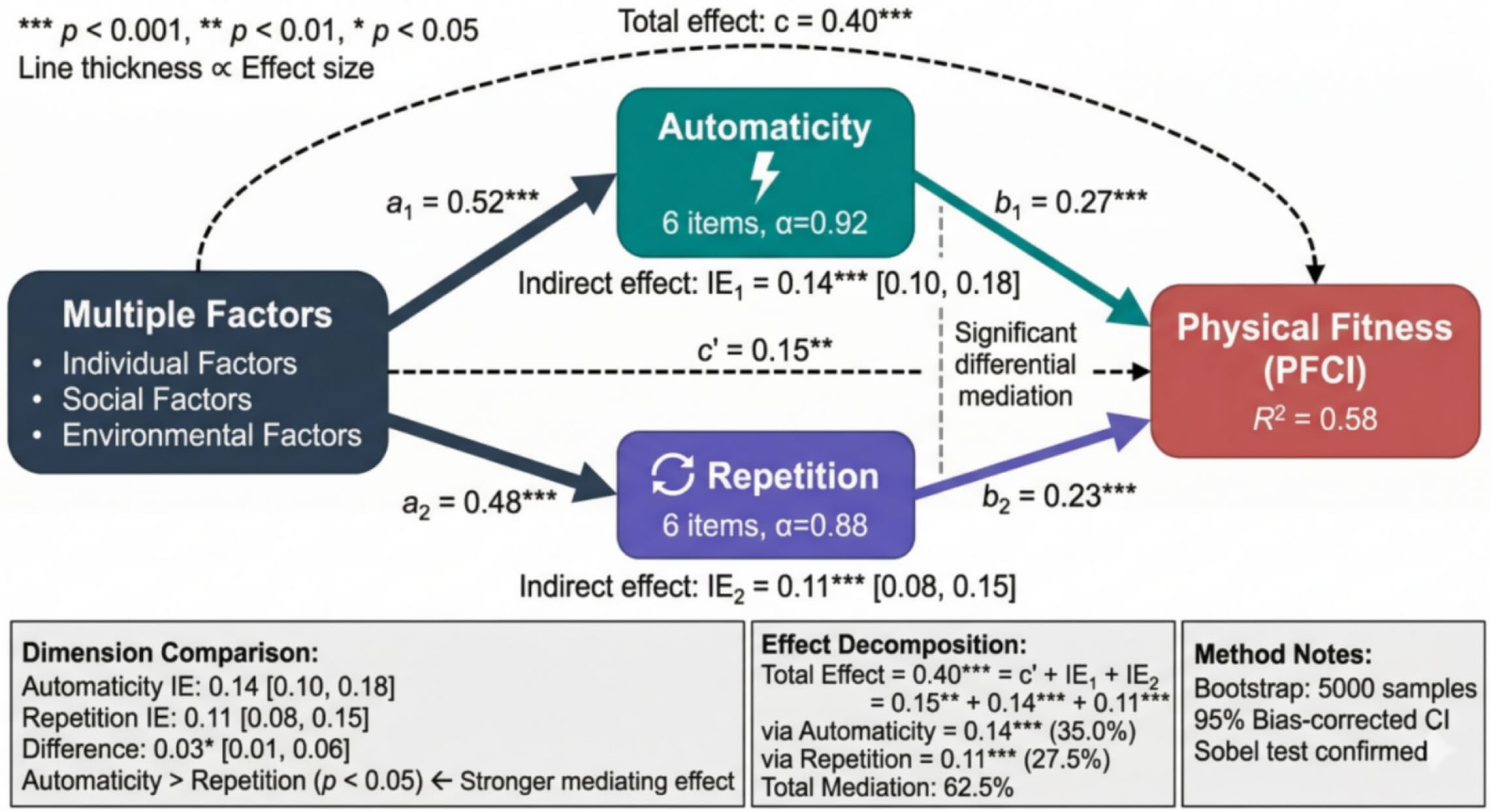
Dual-dimension parallel mediation model of exercise habits.

Multiple mediation analysis further explored the differential mediating effects of the two dimensions of exercise habits (automaticity and repetition). As shown in Figure 10, the parallel mediation model results showed that the mediating effect of the automaticity dimension (*β* = 0.14, *p* < 0.001) was slightly stronger than that of the repetition dimension (*β* = 0.11, *p* < 0.001). The difference test of mediating effects on the two paths showed that the difference was 0.03 (95% CI: 0.01–0.06), reaching statistical significance. This finding suggests that the spontaneity dimension of exercise behavior may be particularly relevant to physical fitness outcomes, and that intervention efforts targeting the automaticity of exercise behavior may deserve attention alongside frequency-based approaches.

### 4.4 Dynamic analysis of tracking data

As the first tier of supporting analyses outlined in Section 3.3, longitudinal methods were employed to examine the temporal dynamics of the primary mediation findings. The latent growth curve model was used to analyze the change trajectories of physical fitness and health and exercise habits and their associations. The model demonstrated good fit to the data (χ^2^/df = 2.08, CFI = 0.968, TLI = 0.962, RMSEA = 0.044, SRMR = 0.039). Model estimation results showed that the mean initial level of physical fitness and health was 73.18 (SE = 0.26, *p* < 0.001), and the linear slope was −1.58 (SE = 0.09, *p* < 0.001), indicating an average annual decrease of 1.58 points. There was a significant negative correlation between initial level and change slope (*r* = −0.34, *p* < 0.001), meaning that students with higher initial physical fitness and health levels experienced faster rates of decline. The change trajectory of exercise habits showed a similar pattern, with an initial level of 4.79 (SE = 0.04, *p* < 0.001), a linear slope of −0.32 (SE = 0.02, *p* < 0.001), and a quadratic term coefficient of −0.04 (SE = 0.01, *p* < 0.01), indicating that the decline rate accelerated in the later period.

**Figure 11:**
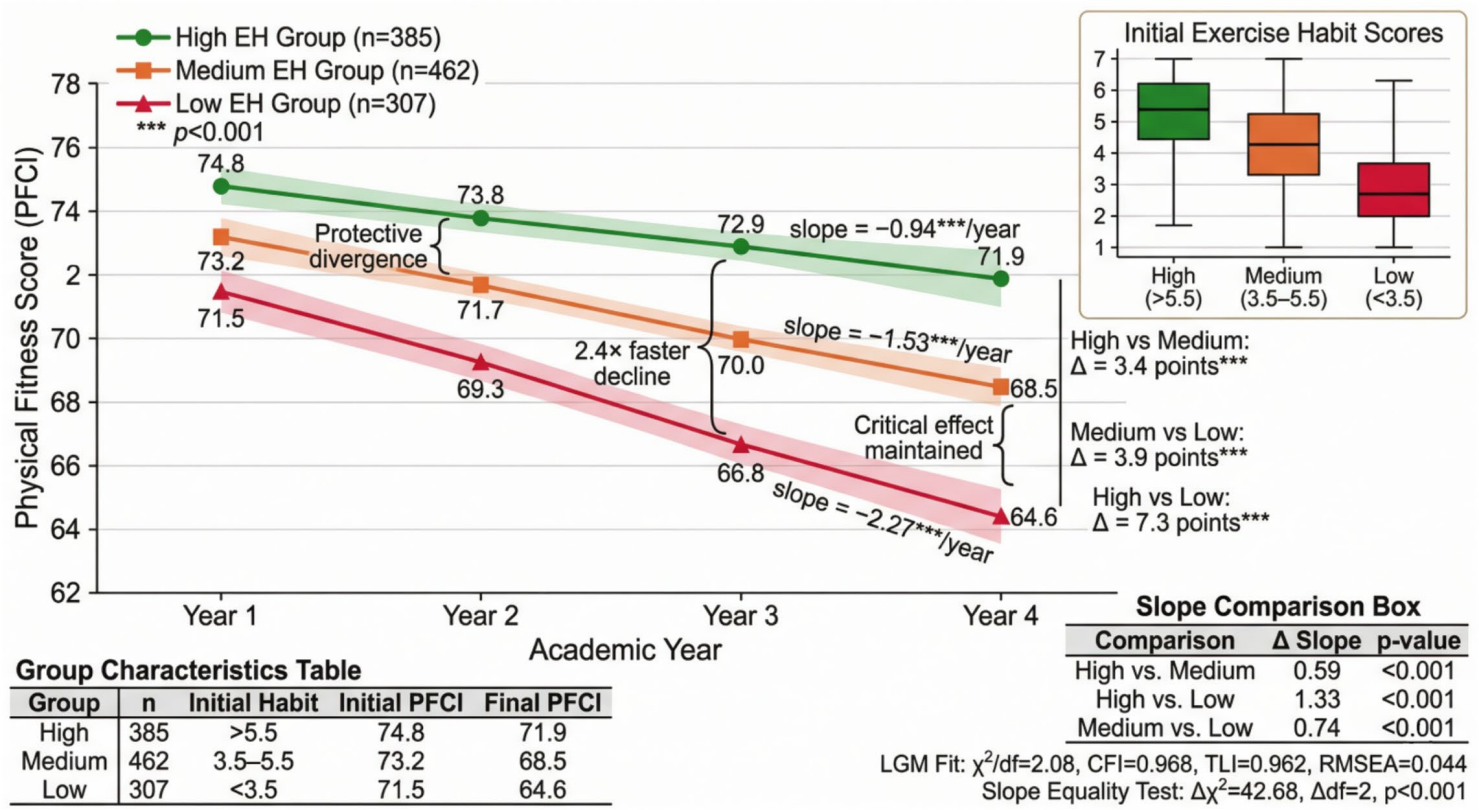
Combined diagram of physical fitness and health change trajectories for different exercise habit intensity groups.

As shown in Figure 11, the combined diagram displays differences in change trajectories among different subgroups. According to initial exercise habit intensity, the sample was divided into three groups: high, medium, and low. The high exercise habit group (*n* = 385) had the smallest decrease in physical fitness and health (slope = −0.94, *p* < 0.001), the medium group (*n* = 462) had a moderate decrease (slope = −1.53, *p* < 0.001), and the low exercise habit group (*n* = 307) showed the most significant decrease (slope = −2.27, *p* < 0.001). To formally test the significance of inter-group differences, multi-group latent growth curve models were estimated with equality constraints on slope parameters across groups. The results, presented in Table 6, confirmed that the slope differences between all pairs of groups reached statistical significance. The chi-square difference test comparing the constrained model (equal slopes) with the unconstrained model (freely estimated slopes) yielded Δχ^2^ = 42.68 (Δdf = 2, *p* < 0.001), indicating that the rate of physical fitness decline differed significantly across groups defined by initial exercise habit intensity.

**Table 6:** Latent growth curve model: group-specific parameters and inter-group comparisons.

Parameter	High EH group (*n* = 385)	Medium EH group (*n* = 462)	Low EH group (*n* = 307)
Initial level (Mean)	74.83 (SE = 0.42)*	73.21 (SE = 0.38)*	71.52 (SE = 0.49)*
Linear slope (Mean)	−0.94 (SE = 0.11)*	−1.53 (SE = 0.10)*	−2.27 (SE = 0.14)*
Slope variance	0.86*	1.24*	1.78*
Pairwise comparison		Δ Slope	*p*-value
High vs. Medium		0.59	<0.001
High vs. Low		1.33	<0.001
Medium vs. Low		0.74	<0.001
Model fit	χ^2^/df = 2.08	CFI = 0.968	TLI = 0.962
	RMSEA = 0.044	SRMR = 0.039	
Slope equality test	Δχ^2^ = 42.68, Δdf = 2, p < 0.001		

*p* < 0.001. EH = Exercise Habit. SE = Standard Error. Slope equality test compares constrained (equal slopes) vs. unconstrained (free slopes) models. Initial Level and Slope are unstandardized estimates.

Cross-lagged panel analysis further examined the temporal sequence and direction of predictive relationships between exercise habits and physical fitness and health. Table 7 presents cross-lagged path coefficients for three waves of data (T1-T3). The predictive coefficient from exercise habits T1 to physical fitness and health T2 was 0.28 (*p* < 0.001), and the predictive coefficient from physical fitness and health T1 to exercise habits T2 was 0.19 (*p* < 0.01), indicating that the prospective predictive strength of exercise habits on physical fitness and health was stronger than the reverse path. This asymmetry was further pronounced in the path from T2 to T3, with the predictive coefficient of exercise habits rising to 0.35 (*p* < 0.001), while the reverse path coefficient remained at 0.18 (*p* < 0.01). These cross-lagged findings are consistent with the hypothesis that exercise habits temporally precede changes in physical fitness, although the observational nature of the study precludes definitive causal conclusions.

**Table 7:** Cross-lagged panel model path coefficients.

Path	T1 → T2	T2 → T3	Stability coefficient
Exercise habits→Exercise habits	0.68***	0.71***	–
PFCI→PFCI	0.74***	0.76***	–
Exercise Habits→PFCI	0.28***	0.35***	–
PFCI→Exercise habits	0.19**	0.18**	–
Covariance			
T1 Exercise habits↔T1 PFCI	0.42***	–	–
T2 Exercise habits↔T2 PFCI	–	0.46***	–
T3 Exercise habits↔T3 PFCI	–	–	0.51***

Model fit indices: χ^2^/df = 2.16, CFI = 0.974, TLI = 0.969, RMSEA = 0.041. *p < 0.05, **p < 0.01, *p < 0.001. PFCI = Physical Fitness Composite Index.

**Figure 12:**
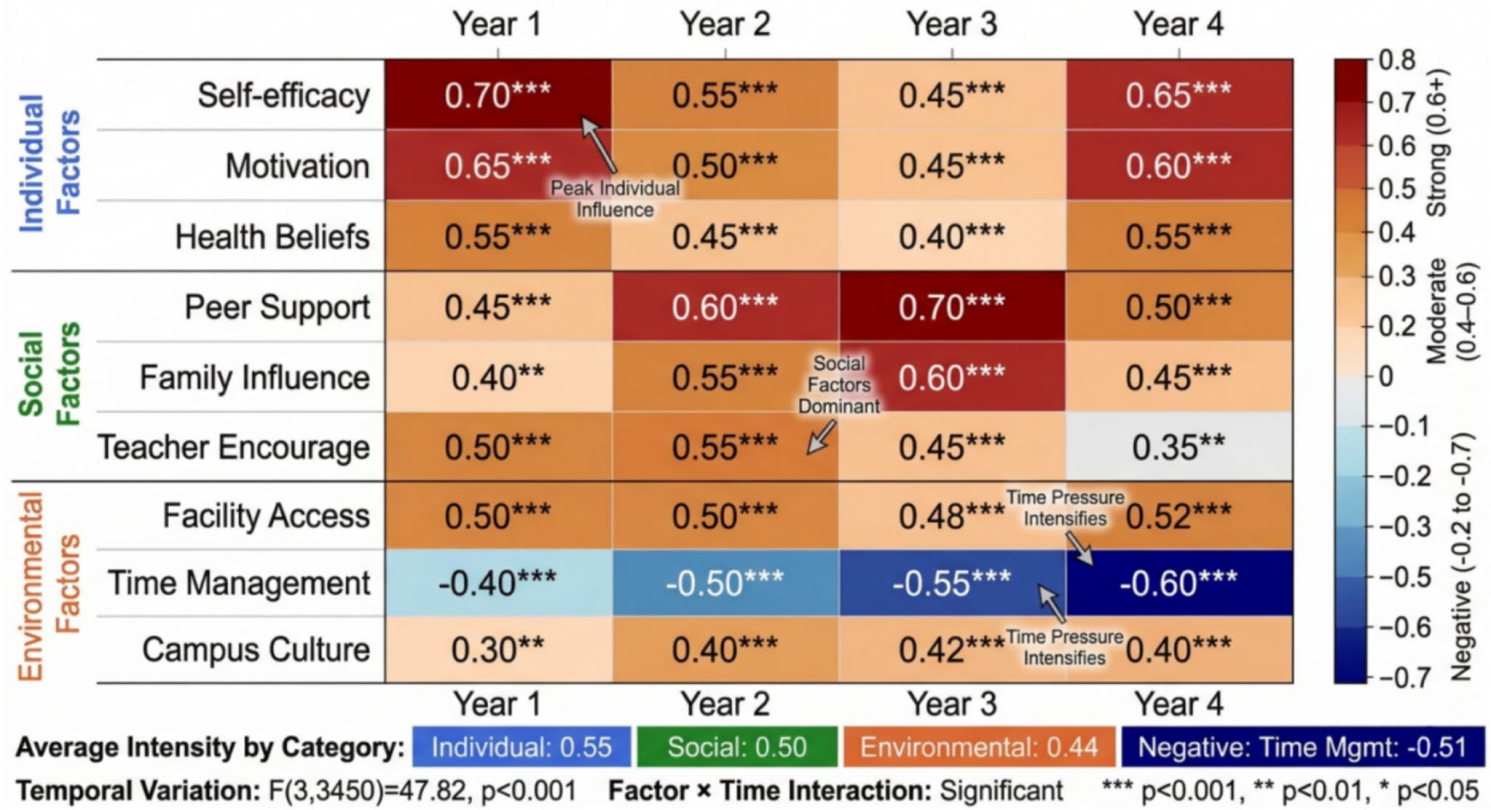
Heat map of influence factor strength during different periods.

Time series analysis identified key turning points and sensitive periods. Piecewise regression results showed that the second semester of sophomore year (fourth semester) was the first turning point for the decline in exercise habits, when students faced major selection and increased academic pressure. The first semester of junior year (fifth semester) was the second critical period, with internship preparation and graduate exam pressure leading to further compression of exercise time. As shown in Figure 12, the heat map intuitively displays the dynamic changes in the influence strength of various factors during different periods. The influence of individual factors was stronger in freshman and senior years, social factors played a greater role in sophomore and junior years, and the influence of environmental factors was relatively stable.

Moderating effect analysis found that gender played a moderating role in multiple paths. The effect of male students’ exercise self-efficacy on exercise habits (*β* = 0.52, *p* < 0.001) was significantly stronger than that of female students (*β* = 0.38, *p* < 0.001), with an interaction term coefficient of 0.14 (*p* < 0.05). The moderating effect of disciplinary background was mainly reflected in the time management pressure path. The negative association between time pressure and exercise habits among medical students (*β* = −0.54, *p* < 0.001) was significantly stronger than that among students from other majors (*β* = −0.37, *p* < 0.001).

Beyond the separate moderating effects of gender and disciplinary background, the two-way interaction of gender × disciplinary background on the path from time management pressure to exercise habits was also examined. The interaction term was statistically significant (*β* = 0.11, *p* < 0.05), indicating that the discipline-related difference in the negative association between time management pressure and exercise habits was more pronounced among male students than among female students. Specifically, male medical students showed the most severe exercise habit decline under high time pressure (simple slope: *β* = −0.62, *p* < 0.001), whereas the discipline-related difference was smaller among female students (Δβ = 0.08, *p* = 0.16).

Additionally, the moderating effect of grade level (academic year) was tested to examine whether the mediating pathway of exercise habits operated differently across the 4 years of college. Multi-group SEM with the sample divided by academic year (freshman, sophomore, junior, senior) revealed that the path coefficient from exercise habits to PFCI increased progressively from 0.31 (*p* < 0.001) in the freshman year to 0.52 (*p* < 0.001) in the senior year. Measurement invariance testing confirmed configural and metric invariance across the four groups (ΔCFI < 0.01), supporting the appropriateness of cross-group comparison. The path from exercise self-efficacy to exercise habits was strongest in the freshman year (*β* = 0.53, *p* < 0.001) and weakest in the junior year (*β* = 0.35, *p* < 0.001), while the path from peer support to exercise habits peaked in the junior year (*β* = 0.51, *p* < 0.001), coinciding with the identified critical period. The indirect effect of exercise self-efficacy on PFCI through exercise habits was 0.16 in the freshman year and 0.18 in the senior year, while the indirect effect of peer support on PFCI through exercise habits increased from 0.10 in the freshman year to 0.21 in the junior year. These grade-level variations suggest that the relative importance of different predictors shifts across the college years, and that targeted approaches may need to be adapted to the specific developmental stage of the students.

**Figure 13:**
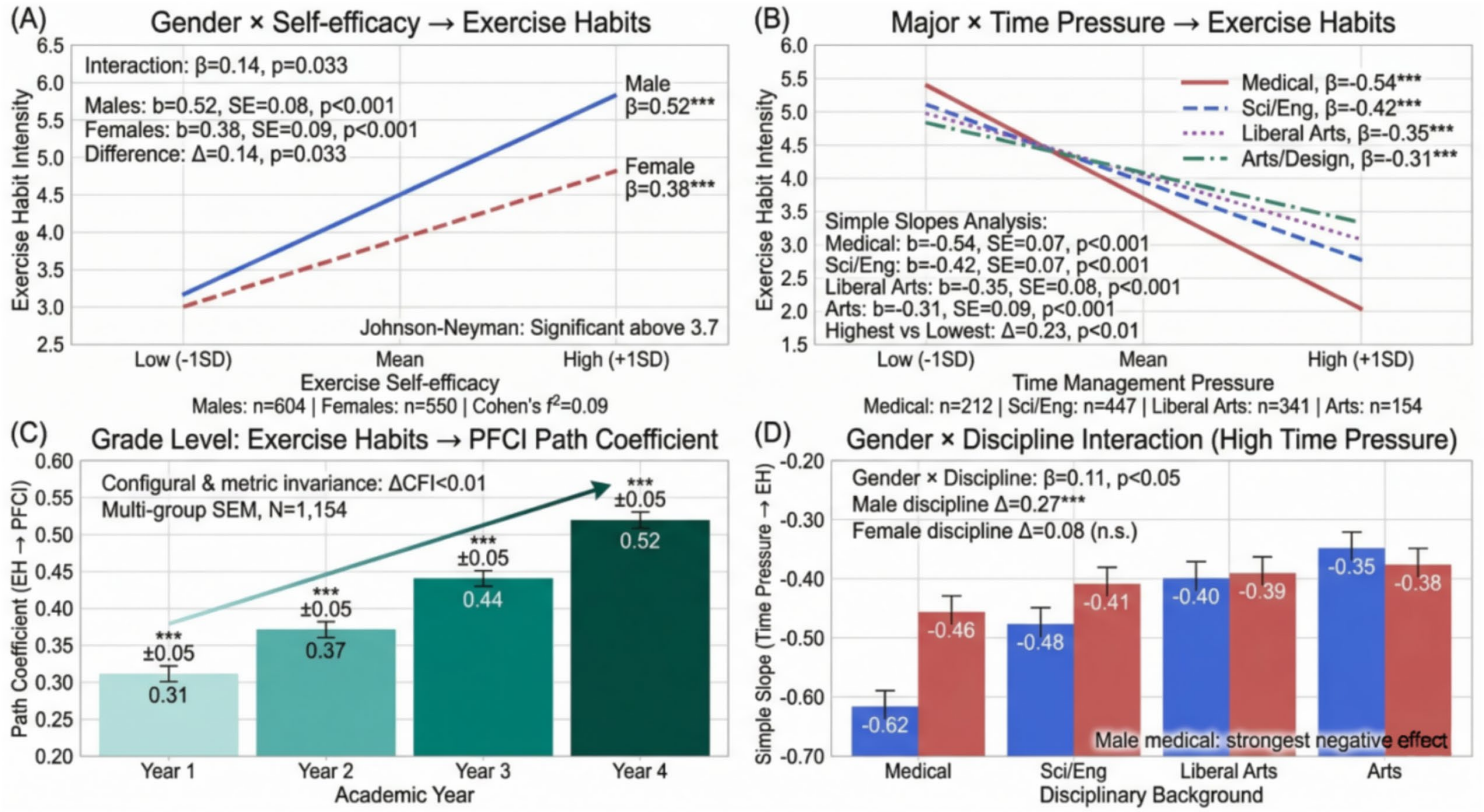
Combined diagram of moderating effects of gender, disciplinary background, and grade level. **(A)** Moderating effect of gender on the relationship between exercise self-efficacy and exercise habit intensity, showing a stronger positive association among males (*β* = 0.52) than females (*β* = 0.38). **(B)** Moderating effect of disciplinary background on the relationship between time management pressure and exercise habit intensity, with medical students showing the steepest negative slope (*β* = −0.54). **(C)** Progressive strengthening of the path coefficient from exercise habits to PFCI across four academic years (from 0.31 to 0.52). **(D)** Two-way interaction of gender × disciplinary background on the simple slope from time management pressure to exercise habits under high time pressure, showing that male medical students experienced the strongest negative effect (*β* = −0.62).

As shown in Figure 13, the moderating effect diagram displays effect differences at different moderator variable levels.

Robustness testing, conducted as the second tier of supporting analyses to verify the primary SEM findings, used multiple methods to assess the reliability of research conclusions. Instrumental variable regression results showed that parental exercise frequency as an instrumental variable satisfied relevance (first-stage *F* = 47.82, *p* < 0.001) and exclusion constraints. The exercise habit predictive coefficient obtained by two-stage least squares estimation was 0.48 (*p* < 0.001), slightly higher than ordinary least squares estimation but with considerable overlap in confidence intervals. Propensity score matching analysis compared physical fitness and health differences between high and low exercise habit groups. The average treatment effect after matching was 8.74 points (*p* < 0.001), which was broadly consistent with the primary regression analysis results. Sensitivity analysis re-estimated the model by gradually removing outliers and influential points. The variation range of core parameter estimates was all within 5%, indicating that the primary findings demonstrate adequate robustness (Table 8).

**Table 8:** Summary of research hypothesis testing results.

Research hypothesis	Hypothesis content	Test result	Effect coefficient	Support level
H1	Individual-level factors positively predict exercise habit formation	Supported	*β* = 0.46***	Fully supported
H2	Social-level factors predict physical fitness and health through exercise habit mediation	Supported	IE = 0.156***	Fully supported
H3	Environmental-level factors have moderating effects on the relationship between exercise habits and physical fitness and health	Partially Supported	Interaction = 0.14*	Partially supported
H4	Exercise habits play a partial mediating role in multiple factors predicting physical fitness and health with group differences	Supported	PM = 39.2–60.9%	Fully supported
H5	Exercise habit stability predicts long-term trends in physical fitness and health changes	Supported	ρ = 0.71–0.76***	Fully supported

***p < 0.001, **p < 0.01, *p < 0.05; IE = Indirect Effect, PM = Proportion of Mediation Effect, ρ = Stability Coefficient. “Supported” indicates that the statistical evidence was consistent with the hypothesized direction and reached significance; it does not imply confirmed causation.

## 5 Discussion and implications

### 5.1 Individual-level factors and exercise habit formation

The positive predictive association between individual-level factors and the formation of exercise habits was supported (H1). Exercise self-efficacy, as the strongest predictor (*β* = 0.46, *p* < 0.001), is consistent with the core viewpoint of social cognitive theory (10). Compared with Sheng et al. (5), who reported that the mediating effect of exercise self-efficacy accounted for 58.5% of the total effect in a cross-sectional study, the present study yielded a somewhat lower mediation proportion (55.0% for the self-efficacy path) but with the advantage of a four-year longitudinal design that provides stronger evidence for the temporal sequence of these associations. The difference in effect sizes may be partly attributable to the fact that Sheng et al. (5) examined a broader range of exercise motivations (including competence motivation), whereas the present study focused specifically on general self-efficacy, motivation, and health beliefs as distinct individual-level predictors. The significant associations of exercise motivation and health beliefs with exercise habits further suggest the importance of internal psychological resources for maintaining regular physical exercise. These findings extend the work of Yu et al. (18, 19), who demonstrated that exercise motivation was associated with self-efficacy through leisure satisfaction and mental toughness, by showing that self-efficacy, motivation, and health beliefs operate as parallel predictors of exercise habits rather than forming a sequential chain.

The grade-level moderation analysis revealed that the predictive strength of exercise self-efficacy on exercise habits was strongest in the freshman year (*β* = 0.53) and weakest in the junior year (*β* = 0.35), suggesting that the role of individual cognitive resources in sustaining exercise behavior may diminish as external pressures intensify during the middle years of college. This temporal pattern implies that interventions aimed at strengthening self-efficacy may be most effective when introduced early in the college experience, before competing academic demands erode students’ confidence in maintaining exercise routines.

### 5.2 Social-level factors and the mediating pathway

The mediating pathway through which social-level factors are associated with physical fitness and health outcomes through exercise habits (H2) received empirical support. The indirect effects of peer support and family influence reached 0.156 and 0.114, respectively, highlighting the potential catalytic role of social support systems in health outcomes. This finding aligns with Yuan et al. (23), who identified a chain mediation pathway from social support through self-efficacy and physical activity to depression outcomes among college students. However, while Yuan et al. (23) focused on mental health outcomes in a cross-sectional design, the present study demonstrates that similar social support mechanisms may also operate in the domain of physical fitness outcomes over a longer time horizon. Notably, the proportion of mediation through exercise habits was higher for the peer support path (60.9%) than for the family influence path (40.7%), which is consistent with the developmental context of college students who are increasingly autonomous from family influence and more responsive to peer norms. Among college students, the demonstration effect and identification mechanism of peer groups appear to be particularly relevant to shaping exercise behavior (24).

A particularly noteworthy finding is that peer support emerged as the dominant predictor during the sophomore-to-junior transition period, with the indirect effect of peer support on PFCI through exercise habits increasing from 0.10 in the freshman year to 0.21 in the junior year. This temporal shift coincides precisely with the critical turning points identified in the longitudinal analysis, suggesting that as individual-level cognitive resources (such as self-efficacy) become less influential during periods of elevated academic pressure, social support networks may compensate by providing external motivation and accountability for maintaining exercise routines.

### 5.3 Environmental-level factors and moderating effects

The moderating effect of environmental-level factors on the relationship between exercise habits and physical fitness and health (H3) received partial support in the facility accessibility and time management dimensions. The negative association between time management pressure and exercise habits (*β* = −0.21, *p* < 0.001) is consistent with findings from Brown et al. (2), whose systematic review using the COM-B model identified opportunity-related barriers (including time constraints and environmental access) as critical domains affecting university students’ physical activity. However, the present study extends this finding by quantifying the specific strength of the time management pathway and demonstrating that its negative association persists after controlling for individual and social factors. The moderating effect of disciplinary background on this pathway—with medical students showing a notably stronger negative association (*β* = −0.54) compared to other disciplines (*β* = −0.37)—is a novel contribution that was not examined in previous systematic reviews. This finding provides nuanced evidence that the academic burden-exercise trade-off is not uniform across disciplines, and that students in academically intensive programs may require targeted support.

Furthermore, the two-way interaction of gender × disciplinary background revealed that the discipline-related difference in the negative association between time management pressure and exercise habits was more pronounced among male students than among female students. Male medical students exhibited the strongest negative association (simple slope: *β* = −0.62), while the discipline-related difference among female students was not statistically significant (Δ*β* = 0.08, *p* = 0.16). This pattern may reflect gender-specific coping strategies under academic pressure: male students in demanding programs may be more likely to reduce exercise as a time-saving strategy, whereas female students may maintain more stable (though potentially already lower) exercise patterns regardless of disciplinary demands. This interpretation, however, requires further investigation through qualitative or mixed-methods research.

### 5.4 Group differences in mediation effects

The partial mediating role of exercise habits in the process through which multiple factors are associated with physical fitness and health (H4) was supported, with the proportion of indirect effect to total effect ranging from 39.2 to 60.9%, and showing significant group difference characteristics. The cross-group analysis revealed that in the male group, the indirect effect of exercise self-efficacy through exercise habits (0.28) was higher than that in the female group (0.19). This gender difference is broadly consistent with Yu et al. (18, 19), who found that the moderating effect of grit on the self-efficacy–physical activity relationship was more prominent in males, suggesting that males may rely more heavily on cognitive confidence mechanisms to maintain exercise behavior. The present study extends this by demonstrating that the gender difference in mediating pathways persists across a four-year tracking period rather than being a cross-sectional snapshot. The negative association between time pressure and exercise habits among medical students (−0.54) was significantly stronger than that among students from other majors (−0.37).

The grade-level moderating analysis revealed that the mediating pathway through exercise habits strengthened progressively across academic years, with the path from exercise habits to PFCI increasing from 0.31 in the freshman year to 0.52 in the senior year. This progressive strengthening suggests that exercise habits become increasingly consequential for physical fitness as students advance through college, potentially reflecting the cumulative nature of both exercise-related physiological adaptations and detraining effects over time. Students who maintain consistent exercise habits may experience compounding fitness benefits, while those whose habits deteriorate may face accelerating fitness losses—a pattern consistent with the dose–response relationship between physical activity and health outcomes documented in the broader epidemiological literature (37).

### 5.5 Longitudinal dynamics and critical transition periods

The predictive association between exercise habit stability and long-term changes in physical fitness and health (H5) revealed by four-year longitudinal data has important practical implications. The annual decrease in physical fitness and health among students with high initial exercise habit intensity (0.94 points) was significantly lower than that in the low exercise habit group (2.27 points). Compared with Tang et al. (1), who tracked 634 medical students and documented a significant downward trend in physical fitness, the present study extends these findings by demonstrating that the rate of decline varies substantially depending on baseline exercise habits, and that this differentiation is evident across all disciplines rather than only among medical students. Cross-lagged analysis further supported the temporal precedence of exercise habits in prospectively predicting physical fitness and health (path coefficient increased from 0.28 for T1 → T2 to 0.35 for T2 → T3). It is important to note that while these cross-lagged findings are consistent with the hypothesis that exercise habits temporally precede and are prospectively associated with physical fitness changes, the observational nature of the data means that definitive causal conclusions cannot be drawn. Unmeasured confounders or reciprocal causal processes that operate on timescales not captured by the annual measurement intervals could potentially explain part of the observed associations. This cumulative association emphasizes the potential value of establishing stable exercise habits in the early college years. At the same time, two key turning points (second semester of sophomore year and first semester of junior year) were identified, providing a more precise time window for formulating phased intervention measures. The grade-level moderation findings further contextualize these turning points by showing that peer support becomes the dominant predictor precisely during the sophomore-to-junior transition, suggesting that social-level interventions may be particularly timely during this critical period.

### 5.6 Limitations

This study has several limitations that should be acknowledged. First, all participants were recruited from a single comprehensive university in northern China. While this design ensured uniform policy conditions and facilitated longitudinal tracking, it limits the generalizability of the findings to other types of institutions (e.g., specialized universities, vocational colleges, private universities), other geographic regions within China, or other countries with different educational systems and campus cultures. In particular, the institutional environment—including campus infrastructure, physical education curriculum requirements, and student demographics—may differ substantially across university types, and the observed associations between exercise habits and physical fitness could vary accordingly. Extending these findings to other institutional contexts requires caution and further investigation through multi-institutional studies.

Second, exercise habits, self-efficacy, exercise motivation, and other psychological variables were measured through self-report questionnaires, which are subject to recall bias, social desirability bias, and the potential for common method variance. Although Harman’s single-factor test and confirmatory factor analysis comparison suggested that common method bias was not a serious concern, future studies would benefit from incorporating objective measures of physical activity (such as accelerometers or wearable devices) to complement self-report data and provide more precise behavioral measurement.

Third, despite the longitudinal design and the use of cross-lagged panel analysis and instrumental variable methods to address endogeneity, this study remains observational in nature. The statistical mediation effects identified here reflect predictive associations rather than experimentally confirmed causal mechanisms. The cross-lagged findings are consistent with the temporal precedence of exercise habits, but unmeasured confounders (such as personality traits, chronic health conditions, or socioeconomic factors not captured by the current measures) could potentially account for part of the observed associations. Experimental or quasi-experimental intervention studies are needed to establish whether changes in exercise habits causally lead to improvements in physical fitness outcomes.

Fourth, as discussed in the theoretical framework section, policy-level factors (such as physical education curriculum design, credit requirements, and health promotion programs) were not included in the empirical analysis because all participants were subject to the same institutional policies. However, policy-level factors may play an important role in shaping exercise behavior, and multi-institutional studies that capture policy-level variation are needed to fully evaluate the contribution of institutional and systemic factors. Future research involving samples from universities with differing physical education requirements, facility provisions, and health promotion initiatives could more effectively examine the role of policy-level factors.

Fifth, the annual measurement intervals may not have captured shorter-term fluctuations in exercise behavior and physical fitness. Exercise habits may exhibit seasonal variation, exam-period disruptions, or event-driven changes that occur on weekly or monthly timescales. Finer-grained measurement (e.g., semester-level or monthly tracking, or ecological momentary assessment) could provide additional insight into the dynamics of habit formation and decline, and could help identify the precise timing and triggers of exercise habit disruptions more accurately than annual assessments allow.

### 5.7 Theoretical contributions and practical implications

The theoretical contributions of this study are reflected in three aspects. First, expanding exercise habits from simple behavioral frequency to a composite concept containing two dimensions of automaticity and repetition deepens the understanding of the essential characteristics of exercise habits. The finding that the indirect effect through the automaticity dimension (0.14) was slightly stronger than that through the repetition dimension (0.11) suggests the dual mechanism of habit formation involving both cognitive and behavioral components. This finding extends the work of Wang et al. (4), who focused primarily on the demand–satisfaction pathway of habit formation, by demonstrating that the automaticity dimension may play a more critical role than simple behavioral repetition in the association between multi-level factors and physical fitness outcomes. Second, using a four-year longitudinal tracking design and cross-lagged panel analysis method addresses the limitations of most existing studies that rely on cross-sectional designs, providing stronger evidence for the temporal ordering of associations between variables rather than simple correlations, and offering dynamic perspective empirical evidence for health behavior change theory. Third, the multi-level theoretical framework integrating the social ecological model, theory of planned behavior, and habit formation theory transcends the explanatory limitations of single theoretical perspectives, systematically examining the predictive relationships among individual, social, and environmental multi-level factors, providing an integrative paradigm for complex health behavior theoretical modeling.

Based on the findings of this study, the following targeted strategies may be considered for supporting college students’ physical fitness and health, though their effectiveness would need to be verified through future intervention studies.

Regarding individual-level interventions, the strong predictive role of exercise self-efficacy suggests that physical education curricula could incorporate mastery experience design, in which exercise tasks are progressively structured so that students frequently experience success at manageable difficulty levels, thereby strengthening their confidence in maintaining exercise routines. Specifically, physical education teachers could implement goal-setting modules at the beginning of each semester in which students set personalized, incremental fitness targets (e.g., improving 800/1000-meter run time by 5 s per month) and receive structured feedback on their progress. Similar progressive goal-setting interventions have been implemented in university physical education programs and have shown positive associations with exercise adherence over one-semester periods (39), suggesting that such approaches are feasible within existing curricular structures without requiring additional staffing or infrastructure. Additionally, brief motivational counseling sessions (15–20 min) could be integrated into freshman orientation programs to help students articulate their personal health values and connect them to specific exercise plans. Given that the present study found exercise self-efficacy to be most influential during the freshman year, timing these sessions at the point of university entry may maximize their potential impact.

Regarding social-level interventions, the finding that peer support exhibited the strongest indirect effect among social factors (mediation proportion of 60.9%) suggests that peer-based approaches may be particularly promising. Universities could establish dormitory-level or class-level exercise partner matching programs in which students are paired with exercise companions based on shared interests and compatible schedules. Such programs could be coordinated by class advisors or student affairs offices, leveraging existing dormitory management structures and class meeting systems to minimize additional administrative burden. Student sports clubs and intramural activity programs could be expanded to lower the entry barrier for non-athletic students, with an emphasis on recreational and social activities (e.g., group hiking, casual sports tournaments, fitness challenges with team-based scoring) rather than competitive performance. Given that the present study found the importance of peer support to peak during the junior year transition period, these social programs should be particularly strengthened during the second semester of sophomore year and the first semester of junior year, when exercise habit decline accelerates. A practical implementation approach would be to launch semester-specific “exercise buddy” campaigns timed to these critical periods, utilizing existing class group messaging platforms and student organization networks for outreach and coordination.

Regarding environmental and institutional-level interventions, the significant negative association between time management pressure and exercise habits—especially among medical students—suggests that institutional scheduling and resource allocation deserve attention. Universities could consider incorporating protected time slots for physical activity into the academic schedule, particularly for programs with intensive coursework loads. This could take the form of designating two to three weekly time blocks (e.g., 4:30–6:00 p.m. on weekdays) during which no classes or mandatory activities are scheduled for students in high-pressure programs, a practice already adopted by some Chinese universities under the “阳光体育” (Sunshine Sports) initiative. Extended facility operating hours (e.g., early morning access from 6:00 a.m. and late evening access until 10:00 p.m.) could accommodate students with tight daytime schedules; the feasibility and cost-effectiveness of such extensions could be assessed through a pilot program during one semester to evaluate student utilization rates before committing to permanent changes. Time management workshops specifically designed for students in academically demanding programs could be offered during the sophomore year, timed to coincide with the identified critical transition period. Campus fitness facilities could be made more accessible through low-cost measures such as distributing small exercise equipment (resistance bands, jump ropes) in dormitory common areas or installing outdoor fitness stations along high-traffic campus pathways—interventions that require minimal capital investment and can be implemented incrementally.

Regarding assessment and curriculum reform, the progressive strengthening of the exercise habits–physical fitness pathway across academic years suggests that university physical education systems should extend beyond the typical two-year required curriculum to provide structured exercise support throughout all 4 years. This could include elective fitness courses, semester-based exercise challenges with academic credit, or digital health platforms that track and encourage regular physical activity. Incorporating exercise habit indicators (such as self-reported exercise frequency and automaticity scores) into the physical fitness evaluation system alongside traditional performance metrics could shift the evaluative emphasis from short-term test preparation to long-term behavior change. It should be acknowledged that the feasibility of these specific suggestions depends on institutional resources, administrative support, and student receptivity; pilot implementation with systematic evaluation would be necessary before scaling to institution-wide adoption. Nevertheless, the proposed strategies are designed to work within existing university administrative and curricular frameworks rather than requiring entirely new infrastructure, which may enhance their practical viability.

## 6 Conclusion

Through longitudinal tracking analysis of four-year physical fitness and health data from 1,154 college students at a comprehensive university in northern China, this study systematically examined the pathways through which multiple factors are associated with physical fitness and health via exercise habits. The PFCI showed a year-by-year downward trend over the four-year period, accompanied by a substantial decrease in exercise habit intensity. A significant positive association was observed between these two trends, and the strength of this association increased over time. Exercise habits served as a statistically significant partial mediator in the predictive pathways from individual factors, social factors, and environmental factors to physical fitness and health, with the proportion of indirect effect to total effect ranging from 39.2 to 60.9%, with social factors’ indirect effects through exercise habits being the most prominent. Exercise self-efficacy, peer support, and time management pressure emerged as the most important predictors at the three levels, respectively. Initial exercise habit intensity was significantly associated with long-term change trajectories of physical fitness and health; students with higher initial exercise habit intensity showed a notably slower rate of PFCI decline compared to those with lower initial levels. Cross-lagged panel analysis provided evidence that exercise habits temporally preceded changes in physical fitness, though the observational design precludes definitive causal interpretation. Two critical transition periods—the second semester of sophomore year and the first semester of junior year—were identified as time points at which exercise habit decline accelerated, with grade-level moderation analysis revealing that peer support became the dominant predictor during these periods.

Several limitations should be briefly noted. The sample was drawn from a single institution, core psychological variables relied on self-report measures, and the findings reflect predictive associations rather than experimentally established causal relationships; detailed discussion of these limitations and their implications is provided in Section 5.6. Future research can address these limitations by expanding sample coverage to include multiple institutions across different regions and institutional types, adopting objective measurement methods such as wearable devices to record exercise data, and conducting randomized controlled trials to test whether targeted interventions that strengthen exercise habits lead to measurable improvements in physical fitness outcomes. Additionally, the application potential of digital health intervention technology in exercise habit cultivation warrants further exploration, as mobile health applications and social media-based peer support platforms may offer scalable and cost-effective means of supporting sustained physical activity among college students.

The theoretical framework and empirical findings of this study provide preliminary scientific evidence for advancing the implementation of the Healthy China strategy in the field of higher education, suggesting that exercise habits represent an important factor associated with college students’ physical fitness and health levels. Through multi-level and targeted systematic approaches, universities may be able to help college students establish and maintain healthy lifestyles that benefit them for life. The extent to which these findings generalize beyond the specific institutional context of this study, and the causal nature of the identified pathways, remain important questions for future investigation.

## Data Availability

The original contributions presented in the study are included in the article/supplementary material, further inquiries can be directed to the corresponding author.
